# Imaging of human stem cell-derived dopamine grafts correlates with behavioural recovery and reveals microstructural brain changes

**DOI:** 10.1016/j.nbd.2025.106910

**Published:** 2025-04-13

**Authors:** Stephen J. Paisey, Lucy R. Jones, David J. Harrison, Nicola J. Drummond, Olivia Z. Edwards, Maurice A. Canham, Victoria H. Roberton, Christopher Marshall, Greg Parker, Rachel Hills, Anne E. Rosser, Emma L. Lane, Stephen B. Dunnett, Tilo Kunath, Yaniv Assaf, Mariah J. Lelos

**Affiliations:** a Wales Research and Diagnostic PET Imaging Centre, School of Medicine, Cardiff University, University Hospital Wales Main Building, Cardiff CF14 4XN, UK; b School of Biosciences, Cardiff University, Museum Avenue, Cardiff CF10 3AX, UK; c Centre for Regenerative Medicine, Institute for Regeneration and Repair, University of Edinburgh, Edinburgh EH16 4UU, UK; d Institute for Stem Cell Research, School of Biological Sciences, University of Edinburgh, Edinburgh EH16 4UU, UK; e Independent Imaging Consultant, Museum Avenue, Cardiff CF10 3AX, UK; f School of Pharmacy and Pharmaceutical Sciences, Cardiff University, Redwood Building, King Edward VII Ave, Cardiff CF10 3NB, UK; g School of Biochemistry Neurobiology Biophysics, Faculty of Life Sciences, Tel Aviv University, Israel; h Medicines Discovery Institute, Cardiff University, Cardiff, CF10 3AT, UK

## Abstract

Cell therapy is a promising therapeutic intervention for Parkinson’s disease (PD) and is currently undergoing safety and efficacy testing in clinical trials worldwide. The goals of this project were (1) to determine whether [^18^F]Fluorodopa or [^18^F]Fallypride imaging correlates robustly with functional recovery; and (2) to explore whether diffusion-weighted MR imaging (DWI) could detect graft-induced cytoarchitectural changes in the host brain. hfVM and hESC-derived dopamine precursor cells were transplanted into the 6-OHDA lesioned rat striatum. Tests of motor function and PET and MR imaging were conducted up to 6 months post-transplantation. Our data demonstrate that [^18^F]Fluorodopa imaging identified presynaptic DA synthesis from hfVM and hESC-derived dopaminergic grafts and [^18^F]Fallypride imaging confirmed occupancy and normalisation of D_2_/D_3_ receptor expression in the grafted hemisphere. In hfVM grafted rats, [^18^F]Fluorodopa binding correlated robustly with motor recovery on a range of drug-induced and drug-free behavioural tasks. In hESC-DA grafted rats, improvements in [^18^F]Fluorodopa PET imaging signals preceded recovery of naturalistic motor behaviours. DWI revealed widespread graft-mediated microstructural changes in the rodent brain, which did not identify graft placement, but instead may reflect remodelling of neuroglia. These data further our understanding of the impact of dopaminergic grafts on brain cytoarchitecture and the potential of these radioligands to predict graft efficacy may aid in the translation of therapeutics from preclinical to clinical settings.

## 1 Introduction

Cell replacement therapies are being explored as a method to establish long-term reinnervation of the striatal nucleus with dopami-nergic (DA) neurons, by replacing the nigral DA cells lost during the course of Parkinson’s disease (PD). Early clinical trials using human fetal ventral mesencephalic (hfVM) transplants established proof-of-principle that DA grafts can survive for over 20 years (Kefalopoulou et al., 2014; Li et al., 2016) and, in some patients, induce significant long-term functional improvement (Barker et al., 2013). Currently, novel human pluripotent stem cell-(hPSC-) derived DA cell therapy products (CTP) are being explored as a more clinically suitable cell source, and the first in-patient hPSC trials are underway in the U.S.A., Europe and Asia to establish the safety and efficacy of this approach (Schweitzer et al., 2020; Takahashi and Price-Evans, 2019; Barker et al., 2017; Clark et al., 2024).

As we work towards widespread clinical application of these novel hPSC-derived CTPs, it becomes pertinent to develop sensitive imaging assays to track cell survival, function and the impact of these cells on the host brain long-term. Development of preclinical translational assays would allow closer mapping of preclinical and clinical datasets and should improve the predictive validity and translation of treatments from the laboratory to the clinic. Moreover, the application of novel imaging assays will allow valuable additional information about ongoing graft survival, integration and biological function to be collected in both preclinical and clinical settings.

State-of-the-art positron emission tomography (PET) imaging approaches have been used in preclinical rodent models, both to characterise disease models and to track neural transplants. [^18^F]Fluorodopa has been used to longitudinally track DA loss in unilaterally 6-hydroxydopamine (6-OHDA)-lesioned rats (Kyono et al., 2011; Becker et al., 2017) and to identify increased turnover of DA in a novel SNCA model of Parkinson’s disease (Morley et al., 2023). DA transplants have been imaged using radioligands for presynaptic dopamine transporter (DAT) imaging (e.g. [^18^F]FBCTT and [^18^F]LBT999) (Goggi et al., 2020; Grealish et al., 2014). [^18^F]Fallypride, which labels postsynaptic D_2_/D_3_ receptors, has been the most frequently employed imaging technology for detecting improvements post-transplantation of DA cells (Goggi et al., 2020; Grealish et al., 2014). Interestingly, [^18^F]Fluorodopa was first used recently to successfully assess DA synthesis from rat fetal VM and hESC-derived grafts in rat models of PD, although in these studies neither the number of cells in the grafts nor the relationship to behavioural outcomes was reported (Park et al., 2024; Tseng et al., 2022). In primates, hiPSC-derived DA grafted regions have been identified using T2-weighted magnetic resonance (MR) imaging and [^18^F]fluorothymidine (FLT)-PET, which can be used to detect proliferating cells (Kikuchi et al., 2017).

Here, we employ PET and MR imaging assays in the unilateral 6-OHDA medial forebrain bundle (MFB) lesioned rat model of PD transplanted with either hfVM or hESC-derived cells. We apply sensitive imaging techniques to detect these small grafts in order to (1) determine whether graft detection using [^18^F]Fluorodopa or [^18^F]Fallypride imaging correlates robustly with behavioural recovery; (2) explore whether diffusion-weighted MR imaging (DWI) can detect graft-induced cytoarchitectural changes in the host brain. These data will allow the non-invasive biological characterisation of the grafts in vivo and will directly determine the sensitivity of these methods in predicting the functional (behavioural) impact of the intervention.

[^18^F]Fluorodopa and [^18^F]Fallypride PET were used to visualise presynaptic DA synthesis and postsynaptic D_2_/D_3_ receptor changes, respectively. As the gold-standard radiotracer in the clinic for PD, [18F] Fluorodopa has been employed previously to track hfVM graft survival in patients and remains the benchmark for monitoring novel hPSC-derived grafts (Schweitzer et al., 2020; Barker et al., 2017; Yilong et al., 2010; Cochen et al., 2003; Politis and Piccini, 2012; Sawle et al., 1992; Kordower et al., 1995; Remy et al., 1995; Freed et al., 2001; Olanow et al., 2003; Piccini et al., 1999; Piccini et al., 2000; Madrazo et al., 2019). However, using preclinical rodent models of PD here, we demonstrate successful detection of presynaptic DA activity and post-synaptic DA receptor normalisation in both hfVM and hESC-derived DA grafted rats. Moreover, these data were closely correlated with the behavioural data, validating the ability of both [^18^F]Fluorodopa and [^18^F]Fallypride PET data to predict the functional efficacy of the grafts.

We also used diffusion weighted MR imaging DWI to determine if this approach was sensitive to detect graft survival and integration, as well as to measure the impact of the graft on the wiser host brain architecture. We determined that DWI was not sensitive to microstructural changes within the striatal nucleus induced by the graft itself, but instead this approach revealed more widespread changes in the micro-structure of distal, non-striatal regions in the grafted brain, which aligned with changes in the density of astrocytes, microglia and oligodendrocytes.

Taken together, the data presented here suggest that [^18^F]Fluorodopa and [^18^F]Fallypride PET data can be used to predict graft efficacy and, while DWI is unlikely to be used to detect the graft itself, it is nevertheless sensitive to microstructural changes induced by glial remodelling.

## 2 Materials and methods

### 2.1 Experimental plan

**Fig. 1:**
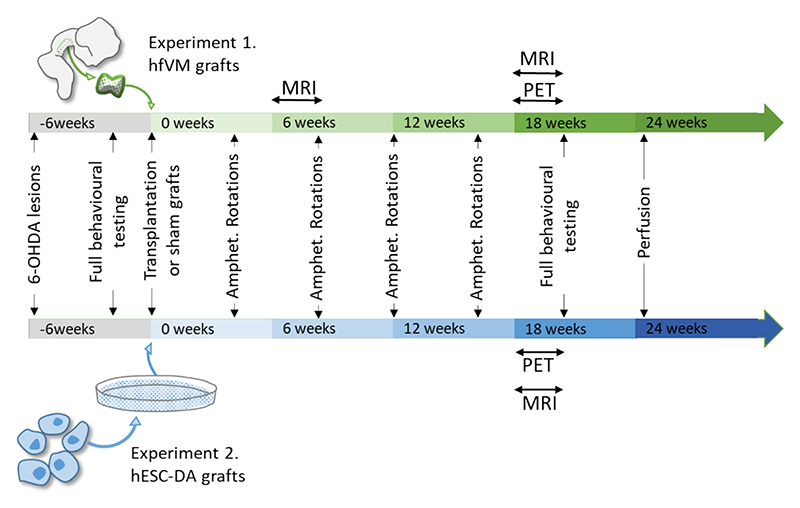
Timeline of the experimental design for Experiment 1 (hfVM grafts) and Experiment 2 (hESC-DA grafts). In Experiment 1, rats received 6-OHDA lesions of the medial forebrain bundle (MFB), then underwent post-lesion behavioural testing to confirm lesion-induced deficits. They subsequently received intrastriatal hfVM grafts or sham grafts, and monthly methamphetamine-induced rotation testing. Initial pilot runs of PET imaging commenced at 15 weeks post-graft, but the main experimental block of PET imaging and MR DWI was undertaken 18–20 weeks post-graft. The broader battery of behavioural testing commenced at 20 weeks post-graft and rats were transcardially perfused at 24 weeks post-graft to harvest tissue for immunohistochemical analysis. In Experiment 2, rats received 6-OHDA lesions of the MFB, then underwent post-lesion behavioural testing to confirm lesion-induced deficits. They subsequently received intrastriatal hESC-derived grafts or sham grafts, and monthly methamphetamine-induced rotation testing commenced. PET imaging and DWI were undertaken between 18 and 20 weeks post-graft. The full battery of hand testing commenced at 20 weeks post-graft and rats were transcardially perfused at 24 weeks post-graft to harvest tissue for immunohistochemical analysis.

Two experiments were conducted to determine whether PET and MR imaging could be used in rat model of PD to visualise authentic hfVM grafts (Experiment 1) and hESC-derived DA grafts (Experiment 2). See Fig. 1 for an experimental timeline.

#### 2.1.1 Experiment 1: hfVM

Twenty rats received 6-hydroxydopamine (6-OHDA) unilateral medial forebrain bundle (MFB) lesions and were tested on a series of motor tasks at 3 and 4 weeks post-lesion. From 1 day prior to graft surgery, all rats in the experiment commenced daily 10 mg/kg i.p. injections of cyclosporine-A immunosuppression. A subset of rats (*n* = 11) received transplants of hfVM tissue to the ipsilateral striatum and the remaining 9 rats received sham transplants, consisting of infusion of vehicle at the same flow rate as was used for the cell transplants. All rats underwent MR imaging (T2-weighted and DWI) at 18–20 weeks post-graft. Subsets of rats (*n*= 6/9 lesion and *n* = 10/11 grafted) underwent [^18^F]Flurodopa PET imaging at 18–20 weeks post-graft. Most rats (*n* = 9/9 lesion and *n* = 7/11 grafted) underwent [^18^F]Fallypride PET imaging at 18–20 weeks post-graft. For both experiments, power calculations were based on data from Grealish and colleagues (Grealish et al., 2014), who used *n* = 6 for [^18^F]Fallypride imaging (effect size 1.32 and power of 0.71), and the requirement to PET image a subset of rats was based on pragmatic considerations, more notably the cost of radioligand production and the logistics of moving rats to/from the imaging facility. Every 4 weeks post-graft, rats were tested on the amphetamine-induced rotation test and at from 20 weeks post-graft, rats were assessed on tests of simple motor function (adjusting steps test, vibrissae-evoked touch test, cylinder) and an apomorphine-induced rotation test. At the end of the experiment, rats were culled via transcardial perfusion and brain tissue was harvested for immunohistochemical analysis.

#### 2.1.2 Experiment 2: hESC-derived DA cells

Twenty rats received 6-OHDA unilateral MFB lesions and were tested on a series of motor tasks at 3 and 4 weeks post-lesion. From 1 day prior to graft surgery, all rats in the experiment commenced daily i.p. 10 mg/kg cyclosporine-A immunosuppression. A subset of rats (*n* = 12) received transplants of hESC-derived DA cells in the ipsilateral striatum. The remaining rats received infusions of vehicle at the same flow rate as was used for the cell transplants. All rats underwent MR imaging (T2-weighted and DWI) at 18–20 weeks post-graft. A subset of rats (*n* = 4/8 lesion and *n* = 6/12 grafted) underwent [^18^F]Flurodopa PET imaging at 18–20 weeks post-graft. A subset of rats (n = 6/8 lesion and n= 6/12 grafted) underwent [^18^F]Fallypride PET imaging at 18–20 weeks post-graft. Every 4 weeks post-graft, rats were tested on the amphetamine-induced rotation test and at 20 weeks post-graft, rats were assessed on tests of simple motor function (adjusting steps test, vibrissae-evoked touch test, cylinder) and an apomorphine-induced rotation test. At the end of the experiment, rats were culled via transcardial perfusion and brain tissue was harvested for immunohistochemical analysis.

### 2.2 Animals

For Experiment 1 (hfVM), 20 female Lister-hooded rats (Charles River, UK) weighing 200-220 g were used and for Experiment 2 (hESC-DA), 20 female Lister-hooded rats (Charles River, UK) weighing 200-220 g were used. All rats were housed in groups of four per cage containing woodchip bedding material, cardboard tube, wooden chewstick and paper nesting material. Food and water were available ad lib at all times. Animals were maintained in an environmentally controlled holding room with a 14:10-h light:dark cycle. Testing was conducted during ‘light’ periods between 9.00 h and 17.00 h. All experiments were conducted in compliance with the UK Animals (Scientific Procedures) Act 1986 under Home Office Licence No. 30/2498 and with the approval of the local Cardiff University Ethics Review Committee.

### 2.3 Cell preparation and differentiation

#### 2.3.1 Preparation of hfVM cells

The hfVM tissues were collected from medical terminations of pregnancy with full donor consent, through the SWIFT human fetal tissue bank (http://www.biobankswales.org.uk/swift-research-tissue-bank/), under UK Human Tissue Authority research licence (no. 12457) held by Cardiff University, and with ethical approval of the project from the Bro Taf local research ethics committee. Gestational age was estimated through ultrasound scan prior to the procedure in combination with measurement of fetal regions, using a validated protocol (Kelly et al., 2011).

The ventral mesencephalon (VM) was harvested from three medical terminations of pregnancy of approximately 9 weeks gestation, with a mean (and standard deviation) crown-rump length of 26.5 mm (± 1.96). VM cell suspensions were prepared as follows: Tissue was incubated at 37 °C for 20 min in protease TryplE Express (Life Technologies Inc.) containing 20 U/ml of an endonuclease, Dornase alfa (Dα) (Pulmozyme, Roche). Tissue was washed by 2 changes of Dulbecco’s minimum eagle medium (DMEM) containing 20 U/ml of Dα. Tissue was mechanically dissociated by trituration in 200 μl of DMEM/Dα using 6–10 strokes with a P1000 Gilson pipettor set at 250 μl, followed by 6–10 strokes with a P200 Gilson pipettor set at the same volume to obtain a quasi-single cell suspension. Following this, two x 2 μl samples were taken from the suspension to quantify cell concentration and for viability assessment using the trypan blue exclusion technique. 8 μl of neat trypan blue (Sigma-Aldrich UK) was added to each 2 μl sample and the resulting 10 μl samples were counted using a haemocytometer on an inverted microscope and 10× objective. The viability for each sample was estimated as >95 %. The cells were then spun down in a centrifuge at 28 *g* for 3 min after which the supernatant was removed and the cells resuspended in fresh DMEM/Dα to a final concentration of 125,000 cells/μl. Cells were kept at room temperature during surgery, and gently resuspended before each filling of the injection syringe.

#### 2.3.2 Preparation of hESC-derived DA cells

Approval for the use of RC17 human embryonic stem cells (hESCs) was granted by the MRC Steering Committee for the UK Stem.

Cell Bank and for the Use of Stem Cell Lines (ref. SCSC13–19). RC17 hESCs were differentiated into dopaminergic neurons at the University of Edinburgh using a previously described protocol (Chen et al., 2019). Briefly, hESCs were plated at 40,000 cells/cm^2^ on Laminin-111-coated plates (L111, 5 μg/ml, Biolamina) in 50 % Neurobasal™ medium (Thermo Fisher Scientific), 50 % DMEM/F12 (Thermo Fisher Scientific), B27 (1:50, Thermo Fisher Scientific), N2 (1:100, Thermo Fisher Scientific) and 2 mM L-glutamine (Thermo Fisher Scientific) with Sonic hedgehog (SHH-C24II, 600 ng/ml, R&D), CHIR99021 (1 μM, Miltenyi Biotec), SB431542 (10 μM, Tocris), LDN193189 (100 nM, Stemgent) and Y27632 (10 μM, Tocris). On day 9 medium was supplemented with FGF8b (100 ng/ml, R&D) and heparin (1 μg/ml, Sigma). A detailed version of the protocol is available at: dx.doi.org/10.17504/protocols.io.bddpi25n. DA progenitor cells were cryopreserved at day 11 of differentiation using an established protocol (Drummond et al., 2020), and transported to Cardiff University. DA cells were thawed and replated at 800,000 cells/cm^2^ on L111-coated plates in Neurobasal medium supplemented with B27 (1:50), 2 mM L-glutamine, BDNF (20 ng/ml, Peprotech), GDNF (10 ng/ml, Peprotech), ascorbic acid (0.2 mM, Sigma), FGF8b (100 ng/ml), heparin (1 μg/ml), and Y27632 (10 μM). At day 16 of differentiation, cells were lifted with Accutase to create a final single cell suspension of 125,000 cells/μl in DMEM.

### 2.4 Surgical procedures

#### 2.4.1 6-OHDA Lesions

Rats were anaesthetised with isoflourane (2–4 % with carrier gases oxygen and nitrous oxide) and placed in a Kopf stereotaxic frame. An injection of 3 μl of a 4 μg/μl/(freebase) solution of 6-hydroxydopamine (6-OHDA, Sigma) in 0.01 % ascorbate saline was infused into the medial forebrain bundle (MFB) over 3 min and the needle left in place for a further 2 min to allow for diffusion of the toxin. The stereotaxic coordinates for the MFB were AP: −4.0 mm, ML: −1.3 mm and DV: −7.0 mm. Rats received Metacam (0.5 mg/kg, sc, Boehringer Ingelheim) for peri-surgical analgesia and 5 ml subcutaneous 0.9 % *w*/*v* glucose saline.

#### 2.4.2 Transplantation surgery

Of the 20 lesion rats in Experiment 1 (hfVM), 11 rats received hfVM transplants and 9 remained as lesion only/sham transplant controls. Of the 20 rats in Experiment 2 (hESC-DA), 12 rats received hESC-DA transplants and 8 remained as lesion only/sham transplant controls, but 1 grafted rat was subsequently culled due to illness unrelated to the experimental procedures.

Rats were anaesthetised with isoflourane (2–4 % with carrier gases oxygen and nitrous oxide) and placed in a Kopf stereotaxic frame. 1 μl suspension of 125,000 cells/μl of hfVM or hESC-DA cells were infused at each of the following striatal sites: (1) AP: +0.5 mm, ML: −.0 mm; (2) AP: +1.2, ML: −2.7 mm and at both sites the DV coordinates were −4.0 and −5.0 mm from dura (500,000 total cells/graft). The toothbar was set to −2.4 mm. Cells were injected at a rate of 1 μl/min and the syringe was left in place for a further 3 min diffusion period. Rats received Metacam (0.5 mg/kg, subcutaneous, Boehringer Ingelheim) for peri-surgical analgesia and 5 ml subcutaneous 0.9 % *w*/*v* glucose saline.

### 2.5 Drugs

#### 2.5.1 Drug-induced rotations

For amphetamine-induced rotations, 2.5 mg/kg methamphetamine hydrochloride (Sigma-Aldrich, UK) was dissolved in sterile saline and injected intraperitoneally (i.p.). For apomorphine-induced rotations, 0.05 mg/kg apomorphine hydrochloride hemihydrate (Tocris, UK) was dissolved in sterile saline and injected subcutaneously.

#### 2.5.2 Immunosuppression

Rats were immunosuppressed daily with 10 mg/kg cyclosporine A i. p. (Sandoz Pharmaceutical, U.K.) starting from the day before graft surgery and throughout the entire post-graft period.

#### 2.5.3 PET imaging drugs

Entacapone (40 mg/kg; Sigma SML0654) was dissolved in a 1:4 solution of DMSO and 20 % cyclodextrin in saline (Sigma H107; (2-Hydroxypropyl)-β-cyclodextrin) and adjusted to pH 7.0 with sodium hydroxide. Entacapone was injected i.p. 90 mins before [^18^F]Fluorodopa imaging. Benserazide (10 mg/kg; Sigma B7283) was dissolved in sterile saline and administered i.p. 30 mins prior to [^18^F]Fluorodopa imaging.

### 2.6 Behaviour

#### 2.6.1 Drug-induced rotations

Lateralised DA depletion was assessed by measuring drug-induced rotations in a bank of 16 automated rotometer bowls (Rotorat, Med Associates, Georgia, VT) modelled after the design of Ungerstedt and Arbuthnott (Ungerstedt and Arbuthnott, 1970) which record the frequency of rotations in both clockwise and anticlockwise directions. Amphetamine-induced rotation was measured after i.p. injection of methamphetamine and behaviour was recorded over a period of 90 min. Amphetamine rotation scores were expressed as net ipsilateral rotations (mean number of ipsilateral rotations minus contralateral rotations (in relation to lesioned side)). At post-lesion baseline, rats that rotated a minimum of 6 times per minute could be included in the study, and in these two experiments, all rats rotated a minimum of 6 times per minute. Apomorphine-induced rotations were recorded for 60 min after s.c. injection of apomorphine. Apomorphine rotation scores were expressed as net contralateral rotations (mean number of contralateral rotations minus ipsilateral rotations (in relation to lesioned side)).

#### 2.6.2 Adjusting steps test

Rats were loosely restrained with one forelimb free and moved across an 80 cm distance of flat bench surface with the weight of the animal resting on the free limb. The number of compensating stepwise adjustments made was recorded for each limb in both a forwards and back-wards direction. Three trials were run per limb per direction and a mean value was calculated.

#### 2.6.3 Vibrissae-induced touch test

The vibrissae test has been used previously to assess the impact of both DA-graft in models of PD (Elabi et al., 2018; Elabi et al., 2022; Elabi et al., 2021) and striatal grafts in models of Huntington’s disease (Lelos et al., 2016; Klein et al., 2013). Animals were loosely restrained with one forelimb free and the vibrissae on the ipsilateral side were brushed against the edge of a bench. A successful reflex reaction was counted when the rat extended the forelimb to touch the bench edge immediately after stimulation of the vibrissae. The vibrissae were brushed against the bench edge 10 times on each side of the head and a mean number of successful reflex actions was calculated per side.

#### 2.6.4 Cylinder test

Rats were individually placed in a Perspex cylinder in front of two mirrors at a 90 degree angle from each other to allow for 360° observation. Each session was video recorded and subsequently scored to determine the mean percentage of weight bearing touches on the sides of the cylinder for both the ipsilateral and contralateral paws after approximately 30 weight-bearing touches.

### 2.7 Imaging

#### 2.7.1 Magnetic resonance imaging

Scanning was conducted using a Bruker Avance II 9.4 Tesla (400Mhz) Magnetic Resonance Imaging (MRI) and Magnetic Resonance Spectroscopy (MRS) system, with 20 cm bore and a gradient strength of up to 400 mTm-1. Rats were anaesthetised with isoflourane (4 % with oxygen as carrier gas) and maintained at 1 % during scanning. Diffusion weighted scans were conducted as follows.

##### 2.7.2 DWI

Diffusion weighted images were acquired using a 2D spin-echo EPI sequence with TR of 12,500 ms, TE of 32.324 ms and no averaging; 3 non-diffusion (b0) weighted images were acquired preceding 30 diffusion weighted images (b = 1000 s/mm2) with optimally distributed gradient orientations (Jones et al., 1999). Field of view was 32 × 24 mm with a 128 × 96 matrix (0.25 × 0.25 mm in plane resolution) across 50 slices of 0.4 mm thickness (no gap). Total acquisition time was 27 min and 30 s.

##### 2.7.3 Synthesis of radiotracers

[^18^F]Fluorodopa and [^18^F]Fallypride were produced in-house to GMP standard via nucleophilic substitution reactions carried out on a Trasis AllinOne universal synthesiser. ^18^F-was produced from ^18^O enriched water via the ^18^O (p,n) ^18^F reaction on an IBA Cyclone 18/9 cyclotron.

##### 2.7.4 [^18^F]Fluorodopa PET imaging

Scanning was conducted in a preclinical Mediso Nanoscan 122S PET/CT system. For practical and pragmatic reasons, a subset of 6 lesion and 10 hfVM grafted rats underwent [^18^F]Flurodopa PET imaging. A subset of 3 lesion and 5 hESC-DA grafted rats also underwent this procedure.

Rats received i.p. injections of entacapone 90 mins prior to scan and benserazide i.p. 30 mins prior to the scan. Rats were anaesthetised with gaseous isoflurane (2–4 % with oxygen as a carrier gas) and secured in the ear bars in the prone position on the Mediso multicell rat bed where anaesthesia was maintained through a nose cone (1.5–2 % isoflurane) and body temperature was maintained at 37 °C by heated air circulating through the bed. Breathing rate was monitored via an integrated pneumatic pressure sensor. A scout CT scan was acquired, and the bed was positioned for a 90 min PET scan centred on the head. 50 MBq of [^18^F]Flurodopa (70-800 μL injection volume) was administered via tail vein immediately before PET scan start. A 2.5 min CT scan was acquired (50KeV, 480 projections) at scan end for anatomical reference and attenuation correction. PET data was reconstructed with Mediso’s proprietary Tera-tomo-3D-algorithm (4 iterations of 6 subsets) with attenuation, random and scatter corrections applied. A 41 frame dynamic time series with frame times of 6x10s;6x30s;11x60s;15x180s;3x600s was prepared as per Kyono et al (Kyono et al., 2011). along with an averaged static image (60–90 min post injection) for VOI and preparation and display purposes.

##### 2.7.5 [^18^F]Fallypride PET imaging

For practical and pragmatic reasons, a subset of 7 lesion and 9 hfVM grafted rats underwent [^18^F]Fallypride PET imaging. A subset of 6 lesion and 6 hESC-DA grafted rats also underwent this procedure. Previous work (Ota et al., 2015) has shown only the final 60 min of a 120 min uptake time is required for pharmacokinetic modelling of [^18^F]Fallypride. Following these methods rats were anaesthetised with gaseous isoflurane (2–4 % with oxygen as a carrier gas) and temporarily transferred to a nose cone where they were injected with 20 MBq [^18^F]Fallypride (30-800 μL injection volume) via tail vein. Rats were returned to the warmed induction chamber (37 °C) where anaesthesia was maintained at 1.5–2 % isoflurane for 50 min. The rats were placed in the the Mediso multicell rat bed in the prone position and secured in the earbars where anaesthesia was maintained through a nose cone (1.5–2 % iso-flurane) and body temperature was maintained at 37 °C by heated air circulating through the bed. Breathing rate was monitored via an integrated pneumatic pressure sensor. A scout CT scan was acquired and then a 60 min PET scan centred on the head was started at 60 min post injection time. A 2.5 min CT scan was acquired (50KeV, 480 projections) at scan end for anatomical reference and attenuation correction. PET data was reconstructed with Mediso’s proprietary Tera-tomo-3D-algorithm (4 iterations of 6 subsets) with attenuation, random and scatter corrections applied. A 12 frame dynamic time series with frame times of 12x300s was prepared along with an averaged static image (100–120 min post injection) for VOI and preparation and display purposes.

### 2.8 Perfusion and immunohistochemistry

#### 2.8.1 Perfusion

Rats were terminally anaesthetised with sodium pentobarbital (150 mg/kg i.p., Euthanal™ 1.0 ml/rat, 200 mg/ml: Merial, Harlow, UK) and sacrificed by transcardial perfusion with 50 ml of 0.01 M phosphate buffer followed by 250 ml of 4 %, buffered (pH 7.4) paraformaldehyde (Sigma-Aldrich). Brains were post-fixed for 4 h in 4 % PFA before being transferred to 25 % sucrose solution in PBS. Tissue was mounted on a freezing sledge microtome and sectioned at 40 μm thickness in a 1:12 series. Sections were stored at –20C in ethylene glycol-based cryoprotectant until they were used for immunohistochemical analysis.

#### 2.8.2 Bright-field Immunohistochemistry

Proteins were visualised according to the following protocol. Free-floating sections were incubated in a quench solution of 10 % methanol, 10 % H_2_O_2_, and dH_2_0. After washing with 0.1 M Tris-buffered saline (TBS), tissue was immersed in 3 % normal serum in 0.1 % Triton X-100 in 0.1 M TBS (TXTBS) for 1 h. Tissue was then incubated in primary antibody. The following primary antibodies were using in this study: anti-tyrosine hydroxylase to visualise DAergic neurons (TH; rabbit, 1:2000, Millipore, AB152); anti-human nuclei (HuNu; mouse, 1:1000, Millipore, MAB1281); anti-glial fibrillary acidic protein to visualise astrocytes (GFAP; rabbit, 1:500, Dako, 20,334); anti-ionised calcium-binding adaptor molecule 1 (Iba-1; rabbit, 1:8000, Wako, 01919741); anti-oligodendrocyte transcription factor 2 to visualise oligodendrocytes (Olig2; mouse, 1:500, Neuromics, RA25081). Primary antibody was incubated with 1 % normal serum in TXTBS overnight at room temperature. Tissue was incubated in secondary antibody diluted 1:200 in TBS with 1 % normal serum for 3 h. After washing with TBS, tissue was immersed in an avidin-biotinylated enzyme complex (Vectastain Elite ABC kit, Vector Laboratories) consisting of 1 % reagent A and 1 % reagent B and 1 % normal serum for 2 h. Tissue was then either stained with the peroxidase substrate 3,3′ –diaminobenzidine (DAB) for 3–5 min (DAB Substrate Kit, Vector Laboratories) or the peroxidase substrate vector SG (Vector SG Substrate Kit, Vector Laboratories), before being mounted on gelatin-coated slides, dehydrated in an ascending series of alcohols, cleared in xylene and coverslipped with di-n-butyl Phthalate in Xylene (DPX) mounting mediums.

#### 2.8.3 Cell counting

Grafts were analysed for tyrosine hydroxylase (TH) content, volume and neurite outgrowth (total, including any cells within the overlying cortex). TH + cells were counted manually across a 1:12 series and total cell number was calculated after applying an Abercrombie correction factor. Volume was measured using Visiopharm Integrator System (VIS, version 4.4.6.9) software on an Olympus Canada Inc. Q-Imaging Microscope. Neurite outgrowth was quantified by sampling 5 areas along the lateral and medial aspects of the graft and counting TH+ neurites at each consecutive 100 μm distance from the graft.

Astrocytes (GFAP+ cells), microglia (Iba1+ cells) and oligodendrocytes/oligodendrocyte precursors (Oligo2+ cells) were counted within defined regions that corresponded to significant alterations on the DWI scans, with the researcher blind to the group allocation of each brain. The insular cortex (bregma 1.0 to −1.0 mm), hippocampus (bregma −1.9 to −6.5 mm), thalamic nuclei (bregma −1.8 to −4.8 mm), midbrain (bregma −4.8 to −6.7 mm), piriform cortex (bregma −2.4 to −4.2 mm) and cingulate cortex (bregma 1.5 to −1.0 mm) were analysed by counting the total number of cells within a defined square region of interest (ROI) applied to the section image. GFAP+ and Oligo2+ cells were counted in both the sham/grafted hemisphere, as well as the contralateral hemisphere. For Iba1, we decided to forego counting the contralateral hemisphere and to instead classify Iba1+ cells by morphology, with Stage 1 (ameboid), Stage 2 (thick, stout processes), Stage 3 (long, thick processes) or Stage 4 (ramified), within the lesion/grafted hemisphere. However, no differences in data between sham vs grafted hemisphere were detected for any of the four morphological stages, and data were instead summed, and presented as total Iba1+ cells.

### 2.9 Image analysis

#### 2.9.1 PET scans

[^18^F]Fallypride PET scans were analysed using Vivoquant 4.0 with the additional modelling module. 3D VOI templates were prepared for each rat from the individual static [^18^F]Fallypride scans containing cerebellum as a tissue reference region (drawn by manual placement of predefined oval shape), skull for alignment purposes (drawn by connected threshold tool from the CT scan) and left and right striatal regions for analysis (drawn by connected threshold tool from the PET scan). Sum intensity values were recorded for the VOIs from the static images and the resultant VOI templates were then applied to the dynamic scan reconstructions and DVR values were calculated for the striatal regions using the logan reference plot workflow in the modelling module of Vivoquant 4.0 with the cerebellum as the tissue reference region.

[^18^F]Flurodopa PET scans were analysed by copying the individual rat VOI templates from the [^18^F]Fallypride scans to the [^18^F]Flurodopa PET/CT scans and manually aligning the templates by matching the skull VOI to the CT scan using the reorientation/registration module of Vivoquant 4.0. Ki values were then calculated for the striatal regions from the dynamic [^18^F]Flurodopa PET scan using the patlak plot workflow in the modelling module of Vivoquant 4.0 using the cerebellum as a reference region.

#### 2.9.2 MR scans

##### 2.9.2.1 DWI analysis

All image processing was performed with an inhouse MATLAB (Mathworks, Natick, MA, USA) pipeline that included the following steps:

1. Image registration (non-linear transformation to correct head motion as well as susceptibility and eddy current induced artifacts).
2. Creating a brain mask from the DWI images using a fast-marching algorithm.
3. DTI calculation to produce fractional anisotropy (FA) and mean diffusivity (MD) maps.
4. Transformation of the FA and MD maps to a digitized atlas space (Paxinos and Franklin, 2001). This process was performed in a two-step procedure (Sagi et al., 2012) in which the scans of each mouse were co-registered (rigid body transformation) with the atlas space. The Paxinos atlas labels (Paxinos & Franklin, 2001) were grouped into 24 areas to allow simple localization of the results with respect to the MRI scan resolution.
5. Following the normalisation process, FA and MD maps were smoothed with a 0.4 mm Gaussian kernel.
6. Cluster size determination for statistical analysis: Permutation tests of the data (pixel randomization) were used to calculate the minimal cluster size to minimize false positive clusters. The minimum cluster size for the noise level in our data was set to k > 15 voxels.

##### 2.9.2.2 Statistics

Voxel based *t*-test was used to reveal areas that underwent significant brain change in FA or MD. Voxels were considered significant following the above mentioned cluster based correction at significance level of *p* < 0.05.

### 2.10 Statistics

Behaviour, histology and PET data were analysed by ANOVA (SPSS v27, IBM) for each experiment. Histology (TH, innervation, volume, Iba1) and simple motor behaviours (apomorphine-induced rotation test, adjusting steps test, vibrissae-induced touch test and cylinder test) and PET data ([^18^F]F-Dopa and [^18^F]Fallypride) were analyse by one-way ANOVA with Group as the between subjects factor. GFAP and Olig2 were analysed by two-way ANOVA with Hemisphere and Group as factors. Amphetamine rotations were analysed by ANOVA with Group (Lesion vs Grafted) and Timepoint (pre-graft to 20 weeks post-graft). One-tailed Pearson’s correlations between behavioural and PET data were also conducted using SPSS.

## 3 Results

### 3.1 Histology and behaviour

#### 3.1.1 Experiment 1 (hfVM)

**Fig. 2:**
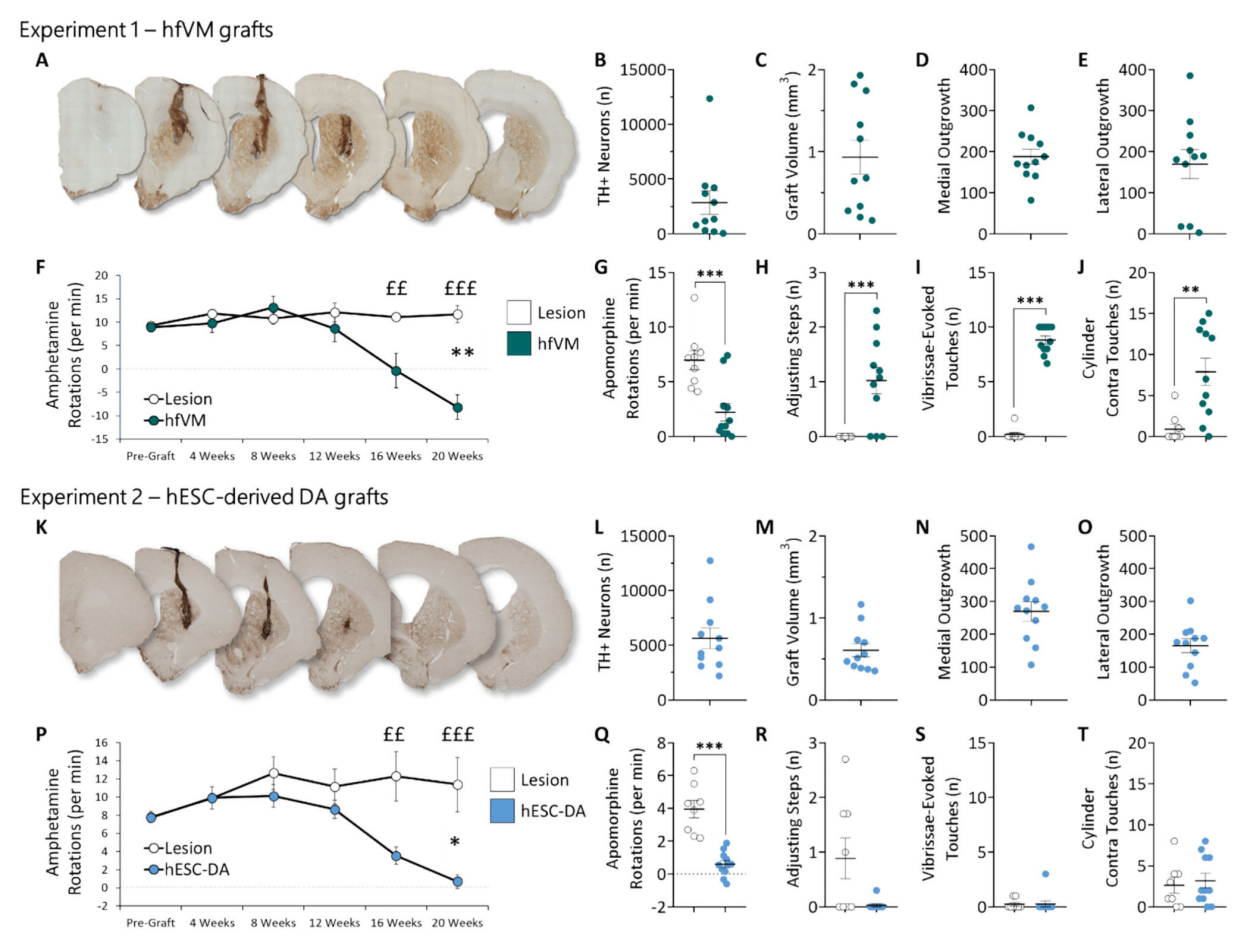
hfVM and hESC-derived DA grafts and post-graft behavioural analysis. Experiment 1: (A) Representative image of a hfVM graft immunostained with TH to visualise DAergic neurons at 20 weeks post-transplantation. (B) Mean TH+ neurons in the hfVM grafts. (C) Mean hfVM graft volume in mm^3^. (D) Mean number of hfVM efferent projections into the host medial striatum. (E) Mean number of hfVM efferent projections into the host lateral striatum. (F) Mean net amphetamine-induced rotations per minute between the pre-graft baseline and 20 weeks post-transplant for sham and hfVM grafted rats, showing gradual loss of rotational bias in grafted rats [Time*Group. F_5,90_ = 11.99, *p* < 0.001; at 16 and 20 weeks post-graft, the effects of group were *p* = 0.005 and p < 0.001, respectively]. Overall, hVM grafted rats rotated less than lesion rats [Group. F_1,18_ = 11.59, p < 0.001], and rotation performance changed over time [Time. F_5,90_ = 9.89, *p* < 0.01]. (G) Mean net apomorphine-induced rotations per minute, showing reduced rotational bias in hfVM grafted rats [F_1,18_ = 16.60, p < 0.001]. (H) Mean number of contralateral adjusting steps at 20 weeks post-transplantation. hfVM grafted rats made significantly more adjusting steps than sham rats [F_1,18_ = 14.49, *p* = 0.001]. (I) Mean number of contralateral vibrassae-evoked touches at 20 weeks post-transplantation. hfVM grafted rats performed significantly more vibrissae-induced touches than sham rats [F_1,18_ = 371.33, p = 0.001]. (J) Mean number of contralateral paw touches in the cylinder test at 20 weeks post-transplantation. hfVM grafted rats performed significantly more touches with the contralateral paw in the cylinder test [F_1,18_ = 13.02, *p* = 0.002] than sham control rats. Experiment 2: (K) Representative image of a hESC-DA graft immunostained with TH to visualise DAergic neurons at 20 weeks post-graft. (L) Mean TH + neurons in the hESC-DA grafts. (M) Mean hESC-DA graft volume in mm^3^. (N) Mean number of projections into the medial striatum emanating from hESC-DA grafts. (O) Mean number of projections into the lateral striatum emanating from hESC-DA grafts. (P) Mean net amphetamine-induced rotations per minute between the pre-graft baseline and 20 weeks post-graft for sham and hESC-DA grafted rats, showing gradual loss of rotational bias in grafted rats [Time*Group. F_5,85_ = 12.05, *p* = 0.001; at 16 and 20 weeks post-graft, the effects of group were *p* = 0.003 and *p* < 0.001, respectively]. Overall, hESC-DA grafted rats rotated less than lesion rats [Group. F_1,17_ = 5.98, *p* < 0.05], and rotation performance changed over time [Time. F_5,85_ = 8.63, p < 0.001]. (Q) hESC-DA grafted also rats rotated significantly less on the apomorphine-induced rotation test [F_1,17_ = 41.79, p < 0.001]. hESC-DA grafts did not improve performance on (R) the adjusting steps test, (S) the vibrissae-induced touch test or (T) the cylinder test at 20 weeks post-transplantation (most F < 1). Data represent means and S.E.M.s. *p ≤ 0.05, **p ≤ 0.01, ***p ≤ 0.001, for the main effect of Group. ^££^p ≤ 0.01, ^£££^p ≤ 0.001, for the effect of Group at each Timepoint.

Representative images of hfVM grafts immunostained for TH to visualise DAergic neurons are presented in Fig. 2 A. hfVM grafts harboured ~2900 TH+ neurons (Fig. 2B) and had a mean volume of just under 1mm^3^ (Fig. 2C). Approximately 200 projections were measured into the medial (Fig. 2D) and lateral (Fig. 2E) host brain striatum. By 16 weeks post-transplant, hfVM grafted rats demonstrated significant improvements in net ipsilateral amphetamine-induced rotational bias relative to sham control rats (Fig. 2F). Moreover, hfVM grafted rats rotated significantly less contralaterally during the apomorphine-induced rotation test (Fig. 2G), made significantly more adjusting steps (Fig. 2H), performed significantly more vibrissae-induced responses (Fig. 2I) and performed significantly more touches with the contralateral paw in the cylinder test (Fig. 2 J) than sham control rats.

### 3.1.2 Experiment 2 (hESC-DA)

Representative images of hESC-DA grafts immunostained for TH to visualise DAergic neurons are presented in Fig. 2 K. hESC-derived grafts harboured ~5600 TH+ neurons (Fig. 2 L) and had a mean volume of ~0.6mm^3^ (Fig. 2 M). Approximately 270 projections were measured into the medial (Fig. 2 N) and 170 into the lateral (Fig. 2O) host brain striatum. By 16 weeks post-transplant, hESC-DA grafted rats demonstrated significant improvements in net ipsilateral amphetamine-induced rotational bias relative to sham control rats (Fig. 2P). The hESC-DA grafted rats also rotated significantly less contralaterally during the apomorphine-induced rotation test (Fig. 2Q). No improvements were evident in the adjusting steps test (Fig. 2R), the vibrissae-induced touch test (Fig. 2S) or the cylinder test (Fig. 2 T).

### 3.2 PET imaging: [^18^F]Fluorodopa

**Fig. 3:**
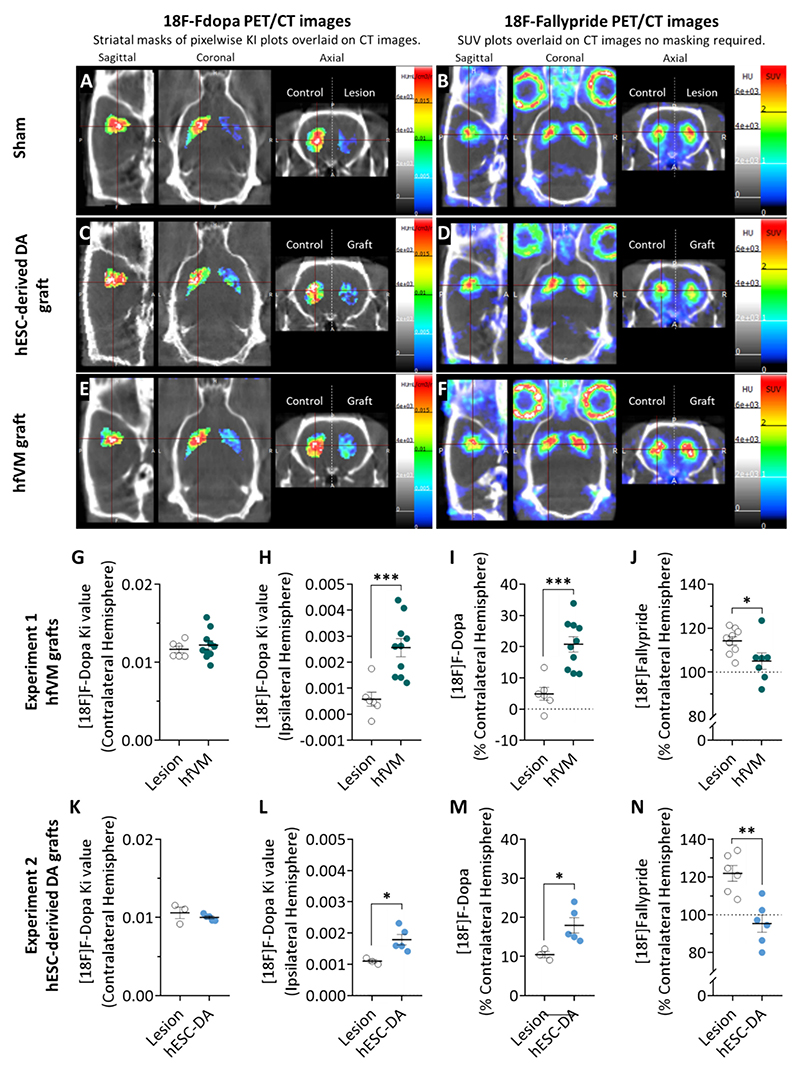
PET imaging of hfVM and hESC-DA grafts. Experiment 1: (A, C & E) Representative pixelwise Ki maps from each group calculated from dynamic [18F] Fluorodopa images by Patlak reference modelling overlaid on the corresponding CT scan. Ki maps were masked to show striatal regions only, to remove background signal and allow thresholds to be set to show uptake in lesioned/grafted regions. (B, D & F) [18F]Fallypride images from the same rats. PET images are presented as SUV maps calculated from the final 20 min of 60–120 min post injection dynamic acquisitions overlaid on the corresponding CT scan. (G) For the hfVM grafted rats, as expected, no group difference in [^18^F]Fluorodopa Ki values was observed in the intact hemisphere (contralateral to lesion) [F_1,15=_0.42, n.s.], while (H) significantly more binding was observed in the lesioned hemisphere where a graft had been implanted [F_1,15_ = 15.65, *p* = 0.001; effect size 1.58]. (I) When the Ki values were expressed as a percentage of the intact hemisphere, a higher percentage of ligand binding was evident for hfVM grafted rats than sham controls [F_1,15_ = 19.82, p = 0.001; effect size 1.79]. (J) hfVM [^18^F]Fallypride imaging data presented as percentage binding relative to intact hemisphere. The reduction in DA production in the lesion rats lead to reduced D_2/_D_3_ receptor occupancy from endogenous DA and increased D_2/_D_3_ receptor expression post-lesion, resulting in higher [^18^F]Fallypride binding. In contrast, the DA production of the hfVM grafts increased endogenous D_2/_D_3_ receptor occupancy and normalised receptor expression leading to a reduction in the [^18^F]Fallypride binding to a level similar to the intact hemisphere only [F_1,15_ = 5.60, *p* = 0.033; effect size 0.90]. Experiment 2: (K) For the hESC-DA grafted rats, as expected, no group difference in [^18^F]Fluorodopa Ki values was observed in the intact hemisphere [F_1,7_ = 1.04, n.s.], (L) while significantly more binding was observed in the lesioned hemisphere where a graft had been implanted [F_1,7_ = 9.32, *p* = 0.02; effect size 2.53]. (M) When Ki values are expressed as a percentage of the intact hemisphere, a higher percentage of ligand binding was evident in hESC-DA grafted rats compared to sham [F_1,7_ = 7.91, p = 0.03; effect size 2.31]. (N) [^18^F]Fallypride imaging data presented as percentage binding relative to intact hemisphere. Sham rats have reduced D_2/_D_3_ receptor occupancy from the lack of natural DA signalling and increased D_2/_D_3_ receptor expression post-lesion, resulting in higher [^18^F]Fallypride binding. In contrast, the DA production of the hESC-DA grafts increased endogenous D_2/_D_3_ receptor occupancy and normalised the receptor expression leading to a reduction in the [^18^F]Fallypride binding to a level similar to the intact hemisphere [F_1,11_ = 18.40, *p* < 0.01; effect size 2.39].

Representative images of [^18^F]Fluorodopa binding from each group are presented in Fig. 3A, C & E, overlaid with the corresponding CT image. The PET images are presented as pixelwise Ki plots (in ml/cm3/ min) calculated in Vivoquant using the patlak graphical reference plot tool with the cerebellum as the reference region. The PET images have also been masked to remove all but the striatal regions using the Ratlas-LH rat brain atlas (Prior et al., 2021) to allow thresholds to be set to show relative uptake levels in the lesioned/grafted side of the brain. Ki values for statistical analyses were calculated using the Vivoquant patlak tool in ROI mode rather than pixelwise mode.

#### 3.2.1 Experiment 1 (hfVM)

As expected, no difference between the raw Ki values obtained from the intact hemisphere of sham and hfVM grafted rats was observed (Fig. 3G). However, significantly higher Ki values were evident in the hfVM grafted hemisphere relative to sham rats (Fig. 3H). Equally, when the Ki values were normalised as a percentage of the intact hemisphere, hfVM had significantly higher binding than sham hemispheres (Fig. 3I). Together, this confirms that [^18^F]Fluorodopa binding was increased at the graft site.

#### 3.2.2 Experiment 2 (hESC-DA)

The results of the hESC-DA grafts mirrored those obtained in the hfVM experiment, with increased [^18^F]Fluorodopa binding at the graft site. That is, no differences between the Ki values obtained from the intact hemisphere of sham and hESC-DA grafted rats were observed (Fig. 3K), but significantly higher Ki values were evident in the hESC-DA grafted hemispheres, relative to sham hemispheres (Fig. 3L). Equally, when the Ki value was normalised as a percentage of the intact hemisphere, higher binding was again identified in the hESC-DA grafted brains (Fig. 3M).

### 3.3 PET imaging: [^18^F]Fallypride

[^18^F]Fallypride binds to D_2_/D_3_ receptors, which are *upregulated* after D depletion. Therefore, a lesion is detected by greater radioligand binding in the affected hemisphere. A mature DAergic transplant would be expected to reduce [^18^F]Fallypride binding, an indication of normalised receptor levels resulting from successful DA replacement. [^18^F] Fallypride images from the same representative rats used for the [^18^F] Fluorodopa images are presented in Fig. 3B, D & F, overlaid over the corresponding CT image. The PET images are presented as SUV values derived from the final 20 min of a dynamic PET acquisition from 60 to 120 min post injection.

#### 3.3.1 Experiment 1 (hfVM)

Analysis of the hfVM graft data normalised to the intact hemisphere revealed a significant difference between hfVM grafted rats and sham rats. This indicates that the DA production of the hfVM grafts increased endogenous D_2/_D_3_ receptor occupancy and normalised receptor expression, leading to a reduction in the [^18^F]Fallypride binding to a level similar to the intact hemisphere (Fig. 3J).

#### 3.3.2 Experiment 2 (hESC-DA)

Similarly, analysis of the hESC-DA graft data normalised to the intact hemisphere revealed a significant difference between hESC-DA grafted rats and sham rats. This change indicates that the DA production of the hfVM grafts increased endogenous D_2/_D_3_ receptor occupancy and normalised receptor expression, leading to a reduction in the [^18^F]Fallypride binding to a level similar to the intact hemisphere (Fig. 3N).

### 3.4 PET imaging correlations

#### 3.4.1 Experiment 1 (hfVM)

For the [^18^F]Fluorodopa, both the raw Ki values and the normalised percentage of binding in the ipsilateral hemisphere correlated strongly with performance on all drug-induced and drug-free naturalistic behavioural tasks (Table 1 and Supplementary Fig. 1). The ratio of [^18^F] Fallypride binding also correlated strongly with functional improvements in the motor tests and the apomorphine-induced rotation test, which is consistent with the effect of apomorphine on D2/D3 receptors. In contrast, [^18^F]Fallypride data did not correlate with amphetamine-induced rotation data. We also correlated the same hfVM PET imaging data ([^18^F]Fluorodopa as raw Ki values or as a percentage of the ipsilateral hemisphere, or [^18^F]Fallypride binding) to TH cells, graft volume and medial/lateral outgrowth. The only significant correlation was between [^18^F]Fluorodopa as a percentage of the ipsilateral hemisphere and TH cells (Pearson’s r: 0.588, *p* < 0.037) and graft volume (Pearson’s r: 0.629, *p* < 0.026).

#### 3.4.2 Experiment 2 (hESC-DA)

For the hESC-derived DA grafts, correlations were identified between performance on the amphetamine-induced rotation task and the ipsilateral [^18^F]Fluorodopa Ki values and the percentage of [^18^F]Fluorodopa, but not with the ratio of [^18^F]Fallypride (Table 1 and Supplementary Fig. 2). This pattern matches that observed with the hfVM grafts. Moreover, significant correlations were identified between performance on the apomorphine rotation test and the ipsilateral [^18^F] Fluorodopa Ki values and the percentage of [^18^F]Fluorodopa and with the ratio of [^18^F]Fallypride. Due to the lack of sufficient recovery in the naturalistic motor tests observed in the hESC-DA grafted rats, it was not relevant to conduct correlational analyses on these data. We also correlated the same hESC-DA PET imaging data ([^18^F]Fluorodopa as raw Ki values or as a percentage of the ipsilateral hemisphere, or [^18^F]Fallypride binding) to TH cells, graft volume and medial/lateral outgrowth. However, no significant correlations were identified.

### 3.5 Diffusion weighted imaging (DWI) reveals widespread changes in cytoarchitecture

#### 3.5.1 Fractional anisotropy

DWI was conducted on rats with hfVM and hESC-derived grafts at 18–20 weeks post-graft to measure microstructural changes in white and grey matter. Fractional anisotropy (FA), which measures the degree of anisotropy in white matter, reflects of the density of fibers, axonal diameter, and myelination. The measure is scalar, with values near 0 indicating isotropic diffusion (i.e. diffusion is unrestricted in all directions) and values closer to 1 indicating perfectly anisotropic diffusion (i.e. diffusion occurs only along one axis).

##### 3.5.1.1 Experiment 1 (hfVM)

We observe FA values indicative isotropic diffusion in most brain regions (range 0.066–0.268; Supplementary Table 1) in both lesion and hfVM grafted rats. After correcting for multiple comparisons, in hfVM-grafted rats, widespread differences in white matter microstructure were observed in the grafted hemisphere (temporal association cortex), bilaterally (medial temporal area, insular cortex, brainstem) and in the ungrafted hemisphere (piriform cortex, ventral lateral cortex, hippocampus, amygdala, thalamus, cerebellum).

##### 3.5.1.2 Experiment 2 (hESC-DA)

As expected, a similar range of FA values were observed in hESC-grafted rats as hfVM (0.074–0.266, Supplementary Table 2). After correcting for multiple comparisons, the hESC-grafted rats presented with changes in the grafted hemisphere (posterior parietal cortex, V1 cortex, ventral medial cortex).

The FA values were increased in all brain regions with significant changes, suggesting more diffusion is occurring along one axis. Taken together, these results suggest that grafts affect white matter microstructure throughout the brain, and increase anisotropy in some brain regions.

#### 3.5.2 Mean diffusivity (MD)

MD represents the overall magnitude of water diffusion in a region or voxel. MD is an inverse measure of the membrane density and values are similar for both grey and white matter. However, it is particularly suited to assessing grey matter because diffusion is isotropic in this tissue. MD is sensitive to the structural integrity of cellular structures (e.g. it detects changes in cellularity, oedema, and necrosis) and this metric increases as microstructural breakdown occurs, such as demyelination and axonal degeneration.

#### 3.5.3 Experiment 1 (hfVM)

In hfVM grafted rats, after correcting for multiple comparisons, reduced MD was evident in the insular cortex in the grafted hemisphere (Supplementary Table 3).

#### 3.5.4 Experiment 2 (hESC-DA)

In hESC-derived DA grafts, bilateral changes were evident (medial temporal area, hypothalamus, thalamus and brainstem), as well as changes in the grafted hemisphere (hippocampus, amygdala) and the ungrafted hemisphere (medial frontal cortex, orbital frontal cortex, frontal association cortex), relative to sham transplanted controls (Supplementary Table 4). In 10 out of 14 brain regions, measures of MD increased. Thus, widespread microstructural changes in MD are detectable after neural transplantation.

### 3.6 Microstructural changes observed on DWI may be due to glial remodelling

To determine whether the changes in the cytoarchitecture within regions distal to the graft site could be related to altered profiles of neuroglia, brain sections were immunostained for astrocytes (GFAP), microglia (Iba1) and oligodendrocytes (Olig2). Four regions were selected that were consistently significantly different between groups (thalamus, hippocampus, piriform cortex, and brainstem), plus the cingulate cortex, differences within which had not withstood correction for multiple comparisons. The results revealed evidence of altered profiles of astrocytes in the thalamus, hippocampus and insular cortex (Supplementary Fig. 3). Oligodendrocytes were altered in the thalamus, piriform cortex and brainstem (Supplementary Fig. 4). Microglia were altered in the cingulate and insular cortices. (Supplementary Fig. 5). These data suggest that DWI may be sensitive to the impact of remodelling of neuroglia on regional cytoarchitecture of the brain.

## 4 Discussion

The aim was to determine whether [^18^F]fluorodopa or fallypride imaging correlate most robustly with functional recovery, and to explore whether DWI could detect graft-induced cytoarchitectural changes in the host brain. We successfully demonstrated detection of small DAergic grafts of both fetal and stem cell origin, using both [^18^F]Fluorodopa and [^18^F]Fallypride. Our data suggest that the hfVM[^18^*F*]Fluorodopa data correlate very closely with functional recovery on tests of both drug-induced behaviours and naturalistic motor performance (adjusting steps and cylinder tests), as well as sensorimotor function (vibrissae-induced touch test) and hESC-DA [^18^F]Fluorodopa data correlate well with drug-induced rotation data. [^18^F]Fallypride data correlated with apomorphine-induced rotations for both hfVM and hESC-DA grafted rats, but not with amphetamine-induced rotations for either group. Finally, we demonstrate that DWI detected changes in brain regions distal from the graft, which may reflect remodelling of neuroglia.

Graft-induced DA release can be detected using [^18^F]Fluorodopa, even prior to behavioural recovery.

In people with PD, [^18^F]Fluorodopa has been used extensively in the clinic to detect the presence of hfVM DAergic neural transplants (Yilong et al., 2010; Freed et al., 2001; Olanow et al., 2003; Piccini et al., 1999; Piccini et al., 2000; Madrazo et al., 2019), and has continued to be the gold-standard radioligand to detect novel stem cell derived DAergic grafts. For example, ongoing clinical trials, such as BlueRock in the USA (NCT04802733), STEM-PD in Sweden and the U.K. (NCT05635409), the Kyoto trial (UMIN000033564), and the Aspen Neuroscience trial in the U.S.A. (NCT06344026) are all using [^18^F]Fluorodopa to detect graft maturation and infer DA content. [^18^F]Fluorodopa requires the aromatic amino acid decarboxylase (AADC) enzyme for conversion to DA, and it is considered more specific to DA presynaptic terminals than vesicular monoamine transporter 2 (VMAT2) and DA transporter (DAT). This is related to the higher specificity of expression of AADC in DAergic presynaptic terminals, whereas VMAT2 and DAT are present within other monominergic projections, or show off-target binding to serotonergic terminals (Politis, 2011; Emond et al., 1997). Despite showing strong specificity for presynaptic DAergic terminals, the [^18^F]Fluorodopa signal can also increase in response to neuroinflammation, which suggests that dual imaging using a radioligand such as [^11^C]PK11195 or [^18^F]GE180 may be suitable to differentiate graft innervation from neuroinflammation (Wilson et al., 2022; Li et al., 2021).

[^18^F]Fluorodopa has been used to image changes in the endogenous DA system in the rodent brain (Kyono et al., 2011; Morley et al., 2023) and to detect dopamine grafts (Park et al., 2024; Tseng et al., 2022). Here we demonstrate that small fetal- or stem cell derived-transplants, harbouring 3000–6000 TH neurons, with volumes of 0.5-1 mm^3^, can be detected by quantifying [^18^F]Fluorodopa binding in a preclinical rat model. These data also correlated positively with tests of drug-induced and naturalistic motor function in hfVM grafted rats, suggesting that this assay can be used both to measure DA graft maturation and to predict the functional efficacy of the graft. Interestingly, hESC-DA grafts could be detected in the brain using [^18^F]Fluorodopa, despite rats showing recovery only on the drug-induced tests and not on the tests of motor or sensorimotor function. This suggests that [^18^F]Fluorodopa is highly sensitive to, and able to detect, small differences in DA synthesis, even before sufficient DA is generated to induce recovery on naturalistic behavioural tests. Establishing this translational assay allows for the direct comparison between preclinical and clinical PET data and may enhance the predictive validity of this model.

### 4.1 Graft detection with [^18^F]Fallypride reveals evidence of neural repair

The radioligand [^18^F]Fallypride binds to post-synaptic D_2_/D_3_ receptors and can be regarded as a measure of neural repair after reinnervation of DAergic projections because it measures receptor occupancy and normalisation after DA replacement. The D_2_/D_3_ receptor antagonist [^11^C]Raclopride has been widely used in clinical trials of DAergic neural transplantation (Wilson et al., 2022), but [^18^F]Fallypride has a higher affinity for D_2_/D_3_ receptors than [^11^C]Raclopride (33 pmol/ L versus 26 nmol/L), as well as a longer half-life (108.9 min versus 20.3 min). In rodent models, [^18^F]Fallypride has been used to measure the impact of stem cell-derived DAergic transplants previously in rodent models of PD (Goggi et al., 2020; Grealish et al., 2014), however our hfVM data are the first to show a positive correlation between [18F] Fallypride and recovery on the apomorphine-induced drug test, on simple motor tests of limb function and on a sensorimotor test. Data from hESC-DA grafts also correlated with apomorphine-induced rotations. Interestingly, we did not observe a correlation with performance on the amphetamine-induced rotation test, a finding that was consistent across both Experiment 1 and 2. This result is consistent with the radioligand being sensitive to post-synaptic remodelling and normalisation, rather than presynaptic DA synthesis. However, [^18^F]Fallypride is subject to competitive displacement from DA, meaning that increased DA in the striatum from the graft can also reduce radioligand binding. Moreover, hESC-DA grafts could be detected in the brain using [^18^F]Fallypride, despite rats showing recovery only on the drug-induced tests and not on the tests of motor or sensorimotor function. This suggests that, at least in our model, [^18^F]Fallypride is highly sensitive to, and able to detect, early changes in DA receptor normalisation and occupancy.

### 4.2 hfVM grafts versus hESC-DA grafts

As noted above, recovery of naturalistic behaviours is evident after transplantation of hfVM grafts, but not observed in rats receiving hESC-derived grafts. While Experiments 1 and 2 were run consecutively, and therefore direct comparison between the experiments is not possible, it is worth considering what may account for these differences. Total TH+ cells are more abundant in hESC-derived grafts and host brain innervation is similar between the groups, if not higher in hESC-derived grafts, making the TH yield or innervation an unlikely explanation for the discrepancy. More feasible is the likelihood that the rate of maturation may differ somewhat between authentic hfVM and hESC-derived cells and/or the complexity of their projections may differ. The latter suggestion is based on the observation that more TH+ neurons in the hESC-derived grafts produce similar innervation levels to the hfVM grafts, suggesting that the hfVM neurons may individually yield more complex processes. However, these hypotheses would need to be tested empirically to ensure that any differences were not simply the result of cell batch variations or transplant procedures.

Moreover, it is worth noting that [18F]Fluorodopa and [18F]Fallypride data distinguished between sham and grafted rats in this study, despite differences in graft sizes and potentially in levels of maturation, between the experiments and conditions. Effect size calculations revealed larger effect sizes overall for PET data generated from hESC-derived grafts (2.31–2.53), than for hfVM grafts (0.90–1.79), which may account for the ability to detect group differences in the hESC-DA experiment, despite smaller group sizes.

#### 4.2.1 DWI reveals altered brain cytoarchitecture post-transplant

**Fig. 4:**
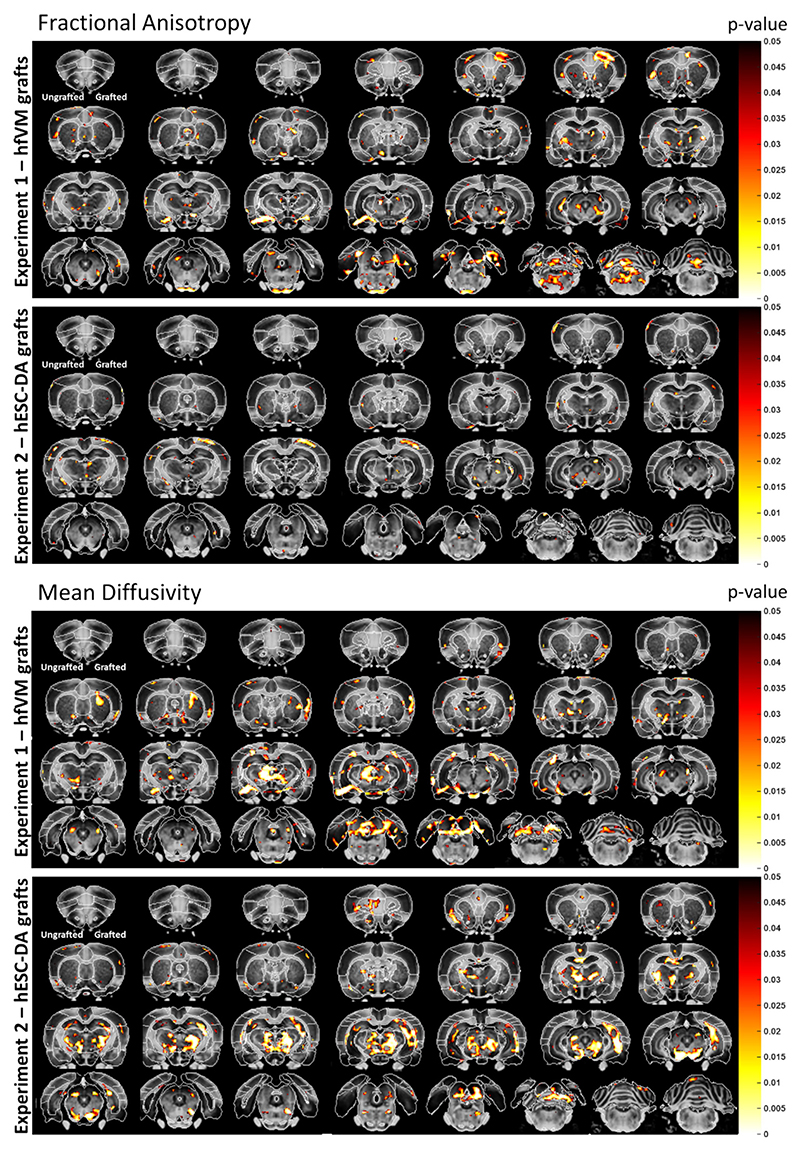
DWI data. Top panel: Fractional anisotropy (FA) data from Experiments 1 and 2. Bottom panel: Mean diffusivity (MD) data from Experiments 1 and 2. *P*-values depicted as heatmap at image edge.

DWI was conducted to determine if this approach was sensitive to microstructural changes in the brain post-graft. Changes in fractional anisotropy (FA), which measures the degree of anisotropy in white matter, may reflect altered density of fibers, axonal diameter, and myelination. Changes in mean diffusivity (MD) may represent alterations in the structural integrity of grey matter cellular structures (e.g. it detects changes in cellularity, odema, and necrosis), although it is also sensitive to changes in white matter. Here, we observed significant differences within restricted voxels in the striatum of grafted rats (e.g. MD for hfVM grafts, Fig. 4), but these differences were not significant for the whole striatal region after correcting for multiple comparisons. This may reflect the significant size of the striatal nucleus and indicate that the relatively small transplants were insufficient to alter the gross architecture of the region. Indeed, as can be seen in Fig. 4, for both MD and FA, significant voxels are evident all around the brain, indicating that sham vs graft differences are widespread, but only some whole brain regions differ significantly between sham and grafted brains once multiple corrections are applied. This threshold may account for some of the differences observed between Experiments 1 and 2 (namely, some of the same brain regions are affected in both experiments, but some regions only meet significance in one experiment).

Our hypothesis was that we would detect differences between sham grafted and grafted rats in the striatum of the grafted hemisphere only. However, we did not observe this, and instead we identified changes in more distal regions of grafted and ungrafted hemispheres. As can be observed in Fig. 2, some graft being present in the overlying temporal cortex could account for some differences in these subregions, which would be interesting and theoretically suggest that DWI could be sensitive to detect grafted areas. However, we do not identify significant differences in the core striatal nucleus, where most of the graft resides, and we do identify differences in a range of other regions that would not be affected by the injection or graft directly. For example, several unanticipated microstructural changes were observed in regions distal to the striatum, including several cortical regions (e.g. insular, piriform and frontal cortices), the thalamus, brainstem and hippocampus. Therefore, we concluded that there must be an alternative explanation that could more parsimoniously explain the range of areas that show differences, and we explored the idea of glial remodelling. To explore the nature of these microstructural changes, we measured the density of neuroglia in select regions of the brain and identified changes in the astrocyte density in the insular cortex, thalamus and hippocampus. Moreover, microglia density was increased in the insular and cingulate corticies and oligodendrocyte density was altered in the brainstem, insular cortex, piriform cortex and thalamus.

The ability of DWI to detect neuroglial changes has been recently reported in mice, with evidence that the DWI is sensitive to changes in morphology and proliferation of astrocytes and microglia (Garcia-Hernandez et al., 2022), as well as recently reported data demonstrating a correlation between MD and microglial cell density based on transcriptomic data (Martínez-Tazo et al., 2024). Thus, widespread remodelling of the neuroglia post-transplantation likely accounts for the microstructural changes detected in distal regions using DWI. It is worth noting that the relatively smaller size of the rat brain results in glial remodelling at regions distal to the transplant site but, within the larger human brain, these glial changes may largely coincide with the site of the transplant (i.e. the putamen or striatal nuclei). Based on our data, DWI is unlikely to be useful biomarkers of graft-mediated recovery and, therefore, we are not necessarily advocating for the use of this approach in the context of neural transplantation. However, DWI may instead reflect a natural immune response to the introduction of the foreign cells, which may be of interest in some studies.

It is worth noting that all rats received two surgeries, namely an MFB lesion, followed by either a sham or graft surgery in the striatum. The effect of the first surgery is controlled for, allowing us to detect the difference between sham and grafted tissues. Clinical application of this methodology would more likely compare the baseline to post-surgical brain. While the implications of this for the DWI signal are as yet unknown in the human brain, it is possible that this approach may yield more broad immune changes, related to both the surgical intervention and the transplant itself.

## 5 Conclusion

Taken together, these data confirm the utility of [^18^F]Fluorodopa and [^18^F]Fallypride as translational assays for preclinical use in detecting presynaptic graft maturation and postsynaptic neural repair and validate their correlation with functional recovery on drug-induced and naturalistic tasks of motor and sensorimotor function. Moreover, we identify widespread changes in neuroglia post-graft that can be detected using DWI. Given the intense interest in stem cell-derived transplantation as a therapeutic approach in PD, these data are important for establishing translational, predictive preclinical assays. Moreover, they represent a non-invasive means of tracking neurobiological mechanisms of recovery longitudinally and are sensitive to presynaptic DA synthesis, post-synaptic receptor remodelling and widespread microstructural changes generated by remodelling of neuroglia.

## Supplementary Material

**Appendix A. Supplementary data**

Supplementary data to this article can be found online at https://doi.org/10.1016/j.nbd.2025.106910.

Supplementary Data

## Figures and Tables

**Table 1:** Correlation between performance on each motor test and PET radioligand binding data for Experiment 1 (top table) and Experiment 2 (bottom table). For Experiment 1, hfVM [^18^F]Fluorodopa data, either as raw Ki binding data or as a percentage of the intact hemsiphere, correlate significantly with performance on most drug-induced and drug-free behavioural tests. The [^18^F]Fallypride data correlated with performance on all drug-free motor tests and apomorphine-induced rotation, but not amphetamine-induced rotations. For Experiment 2, [^18^F]Fluorodopa data correlated with both amphetamine and apomorphine-induced rotation data, while [^18^F]Fallypride data correlated only with apomophine-induced rotation data. Due to the lack of sufficient recovery in naturalistic motor tests observed in the hESC-DA grafted rats, it was not relevant to conduct correlational analyses on these data. *p ≤ 0.05, **p ≤ 0.01, ***p ≤ 0.001.

Scan	Behavioural Assay	Pearson’s r	*p*-value
**Experiment 1: hfVM grafts**			
[18^F^]Fluorodopa:Ki Value	Amphetamine Rotations	– 0.475	*p* = 0.032
	Apomorphine Rotations	– 0.697**	p = 0.001
	Adjusting Steps Test	0.585*	*p* = 0.009
	Vibrissae-Induced Touch Test	0.626**	p = 0.005
	Cylinder Test	0.521*	*p* = 0.019
[18^F^]Fluorodopa: % of intact hemisphere	Amphetamine Rotations	–0.499*	*p* = 0.025
	Apomorphine Rotations	–0.799***	p < 0.001
	Adjusting Steps Test	0.592*	*p* = 0.008
	Vibrissae-Induced Touch Test	0.739**	p = 0.001
	Cylinder Test	0.467	*p* = 0.034
[18^F^]Fallypride: % of intact hemisphere	Amphetamine Rotations	0.052	*p* = 0.424
	Apomorphine Rotations	0.541*	*p* = 0.015
	Adjusting Steps Test	–0.633**	*p* = 0.004
	Vibrissae-Induced Touch Test	–0.560*	*p* = 0.012
	Cylinder Test	–0.523*	p = 0.019
**Experiment 2: hESC-derived DA grafts**			
[18^F^]Fluorodopa: Ki Value	Amphetamine Rotations	– 0.719*	*p* = 0.022
	Apomorphine Rotations	–0.883**	p = 0.002
[18^F^]Fluorodopa: % of intact hemisphere	Amphetamine Rotations	– 0.692	*p* = 0.029
	Apomorphine Rotations	–0.836**	p = 0.005
[18^F^]Fallypride: % of intact hemisphere	Amphetamine Rotations	0.422	*p* = 0.075
	Apomorphine Rotations	0.637*	*p* = 0.013

## Data Availability

Data are freely available upon reasonable request to the corresponding author.
