# Supplementary material for: Imaging of human stem cell-derived dopamine grafts correlates with behavioural recovery and reveals microstructural brain changes
Source: Neurobiol Dis. Author manuscript; Available in PMC 2026 Jun 5. (PMC7619129; doi:10.1016/j.nbd.2025.106910)
Supplement: Supplementary Data [file EMS214108-supplement-Supplementary_Data_.docx]

**Supplementary Files**

***Supplementary Table 1: Functional Anisotropy (FA) values for hfVM grafted rats and sham controls.***

| BRAIN REGION | Hemisphere | Sham (Mean) | hfVM Graft (Mean) | Sham (±SEM) | hfVM Graft (±SEM) | p-value |
| --- | --- | --- | --- | --- | --- | --- |
| Cingulate Cortex | Grafted | 0.144 | 0.144 | 0.005 | 0.004 | 0.94 |
|  | Ungrafted | 0.176 | 0.181 | 0.003 | 0.003 | 0.33 |
| MedTempArea | Grafted | 0.146 | 0.166 | 0.003 | 0.004 | 0.00 |
|  | Ungrafted | 0.154 | 0.171 | 0.003 | 0.005 | 0.02 |
| TempAssoc | Grafted | 0.081 | 0.094 | 0.003 | 0.004 | 0.04 |
|  | Ungrafted | 0.066 | 0.077 | 0.003 | 0.004 | 0.10 |
| Auditory Cortex | Grafted | 0.148 | 0.157 | 0.003 | 0.007 | 0.29 |
|  | Ungrafted | 0.130 | 0.140 | 0.003 | 0.004 | 0.09 |
| Piriform Cortex | Grafted | 0.175 | 0.183 | 0.003 | 0.003 | 0.15 |
|  | Ungrafted | 0.173 | 0.188 | 0.004 | 0.003 | 0.01 |
| Insular Cortex | Grafted | 0.188 | 0.199 | 0.003 | 0.004 | 0.06 |
|  | Ungrafted | 0.204 | 0.218 | 0.002 | 0.005 | 0.04 |
| PPC | Grafted | 0.133 | 0.137 | 0.007 | 0.007 | 0.69 |
|  | Ungrafted | 0.133 | 0.138 | 0.004 | 0.004 | 0.37 |
| S1S2 | Grafted | 0.153 | 0.153 | 0.005 | 0.003 | 0.93 |
|  | Ungrafted | 0.153 | 0.154 | 0.005 | 0.002 | 0.89 |
| V1 | Grafted | 0.086 | 0.095 | 0.002 | 0.003 | 0.08 |
|  | Ungrafted | 0.101 | 0.113 | 0.005 | 0.004 | 0.10 |
| Vlateral | Grafted | 0.074 | 0.099 | 0.012 | 0.008 | 0.09 |
|  | Ungrafted | 0.080 | 0.101 | 0.004 | 0.007 | 0.04 |
| Vmedial | Grafted | 0.108 | 0.110 | 0.008 | 0.006 | 0.90 |
|  | Ungrafted | 0.120 | 0.129 | 0.007 | 0.004 | 0.23 |
| M1M2 | Grafted | 0.169 | 0.161 | 0.008 | 0.003 | 0.26 |
|  | Ungrafted | 0.170 | 0.165 | 0.006 | 0.004 | 0.51 |
| MedialFrontal | Grafted | 0.180 | 0.181 | 0.009 | 0.009 | 0.96 |
|  | Ungrafted | 0.191 | 0.199 | 0.011 | 0.010 | 0.59 |
| OrbitalFrontal | Grafted | 0.163 | 0.182 | 0.009 | 0.011 | 0.23 |
|  | Ungrafted | 0.150 | 0.163 | 0.009 | 0.007 | 0.26 |
| FrontalAssoc | Grafted | 0.110 | 0.099 | 0.018 | 0.009 | 0.55 |
|  | Ungrafted | 0.115 | 0.103 | 0.020 | 0.010 | 0.57 |
| Hippocampus | Grafted | 0.194 | 0.210 | 0.004 | 0.007 | 0.11 |
|  | Ungrafted | 0.180 | 0.192 | 0.002 | 0.004 | 0.02 |
| Amygdala | Grafted | 0.188 | 0.193 | 0.007 | 0.004 | 0.53 |
|  | Ungrafted | 0.195 | 0.215 | 0.004 | 0.005 | 0.01 |
| Striatum | Grafted | 0.251 | 0.251 | 0.004 | 0.006 | 0.93 |
|  | Ungrafted | 0.243 | 0.261 | 0.004 | 0.007 | 0.08 |
| Hypothalamus | Grafted | 0.222 | 0.228 | 0.003 | 0.003 | 0.30 |
|  | Ungrafted | 0.224 | 0.239 | 0.005 | 0.005 | 0.07 |
| Thalamus | Grafted | 0.191 | 0.196 | 0.005 | 0.003 | 0.41 |
|  | Ungrafted | 0.190 | 0.202 | 0.002 | 0.004 | 0.05 |
| Cerebellum | Grafted | 0.132 | 0.160 | 0.007 | 0.010 | 0.07 |
|  | Ungrafted | 0.129 | 0.163 | 0.006 | 0.008 | 0.01 |
| BrainStem | Grafted | 0.185 | 0.217 | 0.008 | 0.008 | 0.02 |
|  | Ungrafted | 0.190 | 0.222 | 0.008 | 0.008 | 0.01 |
| WhiteMatter | Grafted | 0.245 | 0.253 | 0.003 | 0.004 | 0.15 |
|  | Ungrafted | 0.259 | 0.268 | 0.003 | 0.004 | 0.13 |
| Ventricles | Grafted | 0.155 | 0.163 | 0.003 | 0.004 | 0.24 |
|  | Ungrafted | 0.162 | 0.170 | 0.004 | 0.004 | 0.20 |

***Supplementary Table 2: Functional Anisotropy (FA) values for hESC-derived DA grafted rats and sham controls, after correcting for multiple comparisons.***

| BRAIN REGION | Hemisphere | Sham (Mean) | hESC (Mean) | Sham (±SEM) | hESC Graft (±SEM) | p-value |
| --- | --- | --- | --- | --- | --- | --- |
| Cingulate Cortex | Grafted | 0.143 | 0.152 | 0.006 | 0.005 | 0.25 |
|  | Ungrafted | 0.184 | 0.191 | 0.010 | 0.009 | 0.62 |
| MedTempArea | Grafted | 0.158 | 0.165 | 0.007 | 0.009 | 0.52 |
|  | Ungrafted | 0.158 | 0.161 | 0.004 | 0.007 | 0.77 |
| TempAssoc | Grafted | 0.087 | 0.102 | 0.008 | 0.012 | 0.32 |
|  | Ungrafted | 0.074 | 0.080 | 0.004 | 0.010 | 0.55 |
| Auditory Cortex | Grafted | 0.149 | 0.152 | 0.007 | 0.007 | 0.76 |
|  | Ungrafted | 0.131 | 0.139 | 0.006 | 0.008 | 0.48 |
| Piriform Cortex | Grafted | 0.202 | 0.199 | 0.012 | 0.011 | 0.85 |
|  | Ungrafted | 0.194 | 0.185 | 0.006 | 0.008 | 0.40 |
| Insular Cortex | Grafted | 0.217 | 0.206 | 0.008 | 0.007 | 0.30 |
|  | Ungrafted | 0.235 | 0.230 | 0.010 | 0.011 | 0.78 |
| PPC | Grafted | 0.121 | 0.156 | 0.009 | 0.004 | 0.00 |
|  | Ungrafted | 0.129 | 0.135 | 0.005 | 0.004 | 0.36 |
| S1S2 | Grafted | 0.159 | 0.157 | 0.004 | 0.004 | 0.74 |
|  | Ungrafted | 0.164 | 0.160 | 0.004 | 0.006 | 0.66 |
| V1 | Grafted | 0.088 | 0.113 | 0.006 | 0.007 | 0.02 |
|  | Ungrafted | 0.100 | 0.106 | 0.003 | 0.009 | 0.53 |
| Vlateral | Grafted | 0.086 | 0.102 | 0.008 | 0.011 | 0.26 |
|  | Ungrafted | 0.088 | 0.084 | 0.012 | 0.011 | 0.81 |
| Vmedial | Grafted | 0.098 | 0.127 | 0.011 | 0.008 | 0.03 |
|  | Ungrafted | 0.121 | 0.141 | 0.008 | 0.010 | 0.14 |
| M1M2 | Grafted | 0.171 | 0.173 | 0.006 | 0.004 | 0.72 |
|  | Ungrafted | 0.181 | 0.178 | 0.010 | 0.004 | 0.80 |
| MedialFrontal | Grafted | 0.187 | 0.202 | 0.015 | 0.007 | 0.36 |
|  | Ungrafted | 0.214 | 0.215 | 0.017 | 0.006 | 0.97 |
| OrbitalFrontal | Grafted | 0.209 | 0.193 | 0.016 | 0.009 | 0.37 |
|  | Ungrafted | 0.185 | 0.205 | 0.015 | 0.010 | 0.28 |
| FrontalAssoc | Grafted | 0.162 | 0.193 | 0.033 | 0.020 | 0.45 |
|  | Ungrafted | 0.159 | 0.182 | 0.025 | 0.018 | 0.51 |
| Hippocampus | Grafted | 0.209 | 0.208 | 0.005 | 0.005 | 0.88 |
|  | Ungrafted | 0.191 | 0.190 | 0.004 | 0.003 | 0.88 |
| Amygdala | Grafted | 0.199 | 0.200 | 0.009 | 0.009 | 0.97 |
|  | Ungrafted | 0.211 | 0.222 | 0.018 | 0.009 | 0.57 |
| Striatum | Grafted | 0.256 | 0.261 | 0.006 | 0.002 | 0.38 |
|  | Ungrafted | 0.261 | 0.266 | 0.006 | 0.003 | 0.49 |
| Hypothalamus | Grafted | 0.232 | 0.233 | 0.008 | 0.010 | 0.95 |
|  | Ungrafted | 0.235 | 0.234 | 0.006 | 0.003 | 0.88 |
| Thalamus | Grafted | 0.193 | 0.199 | 0.003 | 0.005 | 0.30 |
|  | Ungrafted | 0.204 | 0.205 | 0.006 | 0.003 | 0.91 |
| Cerebellum | Grafted | 0.153 | 0.166 | 0.009 | 0.011 | 0.36 |
|  | Ungrafted | 0.142 | 0.150 | 0.009 | 0.007 | 0.53 |
| BrainStem | Grafted | 0.222 | 0.216 | 0.006 | 0.005 | 0.44 |
|  | Ungrafted | 0.224 | 0.215 | 0.007 | 0.005 | 0.29 |
| WhiteMatter | Grafted | 0.250 | 0.256 | 0.005 | 0.009 | 0.62 |
|  | Ungrafted | 0.264 | 0.266 | 0.008 | 0.011 | 0.87 |
| Ventricles | Grafted | 0.152 | 0.153 | 0.007 | 0.004 | 0.90 |
|  | Ungrafted | 0.162 | 0.164 | 0.008 | 0.006 | 0.82 |

***Supplementary Table 3: Mean diffusivity (MD) values (x10^-3^) for hfVM grafted rats and sham controls, after correcting for multiple comparisons.***

| BRAIN REGION | Hemisphere | Sham (Mean) | hfVM Graft (Mean) | Sham (±SEM) | hfVM Graft (±SEM) | p-value |
| --- | --- | --- | --- | --- | --- | --- |
| Cingulate Cortex | Grafted | 0.084 | 0.089 | 0.003 | 0.002 | 0.25 |
|  | Ungrafted | 0.084 | 0.087 | 0.002 | 0.001 | 0.35 |
| MedTempArea | Grafted | 0.060 | 0.060 | 0.001 | 0.001 | 0.93 |
|  | Ungrafted | 0.060 | 0.060 | 0.000 | 0.001 | 0.86 |
| TempAssoc | Grafted | 0.038 | 0.044 | 0.003 | 0.002 | 0.10 |
|  | Ungrafted | 0.035 | 0.039 | 0.002 | 0.002 | 0.14 |
| Auditory Cortex | Grafted | 0.065 | 0.066 | 0.001 | 0.001 | 0.61 |
|  | Ungrafted | 0.064 | 0.065 | 0.001 | 0.001 | 0.35 |
| Piriform Cortex | Grafted | 0.060 | 0.059 | 0.002 | 0.003 | 0.79 |
|  | Ungrafted | 0.062 | 0.062 | 0.002 | 0.002 | 0.89 |
| Insular Cortex | Grafted | 0.071 | 0.067 | 0.001 | 0.001 | 0.04 |
|  | Ungrafted | 0.074 | 0.073 | 0.001 | 0.001 | 0.34 |
| PPC | Grafted | 0.066 | 0.068 | 0.001 | 0.001 | 0.29 |
|  | Ungrafted | 0.072 | 0.070 | 0.001 | 0.001 | 0.20 |
| S1S2 | Grafted | 0.073 | 0.072 | 0.001 | 0.001 | 0.37 |
|  | Ungrafted | 0.076 | 0.075 | 0.001 | 0.001 | 0.64 |
| V1 | Grafted | 0.048 | 0.051 | 0.003 | 0.002 | 0.40 |
|  | Ungrafted | 0.055 | 0.056 | 0.003 | 0.001 | 0.87 |
| Vlateral | Grafted | 0.041 | 0.050 | 0.007 | 0.002 | 0.23 |
|  | Ungrafted | 0.046 | 0.048 | 0.005 | 0.002 | 0.74 |
| Vmedial | Grafted | 0.052 | 0.053 | 0.003 | 0.002 | 0.83 |
|  | Ungrafted | 0.061 | 0.060 | 0.001 | 0.002 | 0.66 |
| M1M2 | Grafted | 0.065 | 0.068 | 0.002 | 0.002 | 0.35 |
|  | Ungrafted | 0.068 | 0.068 | 0.002 | 0.001 | 0.86 |
| MedialFrontal | Grafted | 0.076 | 0.078 | 0.002 | 0.002 | 0.46 |
|  | Ungrafted | 0.071 | 0.072 | 0.001 | 0.002 | 0.87 |
| OrbitalFrontal | Grafted | 0.058 | 0.055 | 0.001 | 0.002 | 0.18 |
|  | Ungrafted | 0.059 | 0.057 | 0.002 | 0.001 | 0.47 |
| FrontalAssoc | Grafted | 0.030 | 0.027 | 0.004 | 0.002 | 0.48 |
|  | Ungrafted | 0.033 | 0.035 | 0.003 | 0.002 | 0.55 |
| Hippocampus | Grafted | 0.081 | 0.084 | 0.001 | 0.002 | 0.22 |
|  | Ungrafted | 0.080 | 0.083 | 0.001 | 0.002 | 0.09 |
| Amygdala | Grafted | 0.061 | 0.063 | 0.001 | 0.002 | 0.37 |
|  | Ungrafted | 0.061 | 0.065 | 0.002 | 0.001 | 0.11 |
| Striatum | Grafted | 0.079 | 0.079 | 0.001 | 0.001 | 0.93 |
|  | Ungrafted | 0.077 | 0.075 | 0.000 | 0.001 | 0.15 |
| Hypothalamus | Grafted | 0.075 | 0.078 | 0.001 | 0.003 | 0.50 |
|  | Ungrafted | 0.073 | 0.077 | 0.001 | 0.003 | 0.25 |
| Thalamus | Grafted | 0.085 | 0.083 | 0.001 | 0.001 | 0.19 |
|  | Ungrafted | 0.084 | 0.082 | 0.000 | 0.001 | 0.07 |
| Cerebellum | Grafted | 0.057 | 0.062 | 0.002 | 0.002 | 0.08 |
|  | Ungrafted | 0.058 | 0.061 | 0.002 | 0.002 | 0.19 |
| BrainStem | Grafted | 0.076 | 0.075 | 0.001 | 0.001 | 0.72 |
|  | Ungrafted | 0.075 | 0.074 | 0.001 | 0.001 | 0.76 |
| WhiteMatter | Grafted | 0.081 | 0.081 | 0.001 | 0.001 | 0.88 |
|  | Ungrafted | 0.080 | 0.080 | 0.001 | 0.001 | 0.78 |
| Ventricles | Grafted | 0.102 | 0.104 | 0.002 | 0.002 | 0.65 |
|  | Ungrafted | 0.102 | 0.107 | 0.002 | 0.002 | 0.10 |

***Supplementary Table 4: Mean diffusivity (MD) values (x10^-3^) for hESC-derived DA grafted rats and sham controls, after correcting for multiple comparisons.***

| BRAIN REGION | Hemisphere | Sham (Mean) | hESC (Mean) | Sham (±SEM) | hESC Graft (±SEM) | p-value |
| --- | --- | --- | --- | --- | --- | --- |
| Cingulate Cortex | Grafted | 0.087 | 0.089 | 0.003 | 0.002 | 0.28 |
|  | Ungrafted | 0.085 | 0.087 | 0.003 | 0.002 | 0.28 |
| MedTempArea | Grafted | 0.066 | 0.071 | 0.002 | 0.002 | 0.05 |
|  | Ungrafted | 0.065 | 0.069 | 0.001 | 0.002 | 0.04 |
| TempAssoc | Grafted | 0.043 | 0.049 | 0.002 | 0.005 | 0.15 |
|  | Ungrafted | 0.043 | 0.042 | 0.002 | 0.005 | 0.44 |
| Auditory Cortex | Grafted | 0.067 | 0.068 | 0.002 | 0.003 | 0.39 |
|  | Ungrafted | 0.067 | 0.067 | 0.001 | 0.003 | 0.49 |
| Piriform Cortex | Grafted | 0.059 | 0.060 | 0.002 | 0.002 | 0.29 |
|  | Ungrafted | 0.062 | 0.061 | 0.002 | 0.002 | 0.39 |
| Insular Cortex | Grafted | 0.072 | 0.073 | 0.002 | 0.002 | 0.39 |
|  | Ungrafted | 0.077 | 0.076 | 0.001 | 0.002 | 0.41 |
| PPC | Grafted | 0.066 | 0.066 | 0.003 | 0.004 | 0.46 |
|  | Ungrafted | 0.071 | 0.070 | 0.001 | 0.004 | 0.37 |
| S1S2 | Grafted | 0.073 | 0.074 | 0.001 | 0.002 | 0.36 |
|  | Ungrafted | 0.076 | 0.076 | 0.001 | 0.002 | 0.45 |
| V1 | Grafted | 0.052 | 0.055 | 0.003 | 0.004 | 0.24 |
|  | Ungrafted | 0.059 | 0.057 | 0.002 | 0.004 | 0.35 |
| Vlateral | Grafted | 0.048 | 0.054 | 0.003 | 0.005 | 0.16 |
|  | Ungrafted | 0.050 | 0.045 | 0.003 | 0.006 | 0.26 |
| Vmedial | Grafted | 0.053 | 0.058 | 0.003 | 0.004 | 0.17 |
|  | Ungrafted | 0.066 | 0.064 | 0.003 | 0.004 | 0.35 |
| M1M2 | Grafted | 0.074 | 0.073 | 0.001 | 0.001 | 0.45 |
|  | Ungrafted | 0.074 | 0.075 | 0.001 | 0.001 | 0.43 |
| MedialFrontal | Grafted | 0.087 | 0.084 | 0.002 | 0.002 | 0.22 |
|  | Ungrafted | 0.076 | 0.082 | 0.003 | 0.002 | 0.05 |
| OrbitalFrontal | Grafted | 0.060 | 0.069 | 0.004 | 0.004 | 0.07 |
|  | Ungrafted | 0.060 | 0.070 | 0.004 | 0.003 | 0.02 |
| FrontalAssoc | Grafted | 0.042 | 0.057 | 0.005 | 0.006 | 0.06 |
|  | Ungrafted | 0.049 | 0.063 | 0.005 | 0.005 | 0.04 |
| Hippocampus | Grafted | 0.087 | 0.092 | 0.002 | 0.001 | 0.02 |
|  | Ungrafted | 0.087 | 0.087 | 0.002 | 0.002 | 0.48 |
| Amygdala | Grafted | 0.072 | 0.070 | 0.004 | 0.005 | 0.35 |
|  | Ungrafted | 0.071 | 0.064 | 0.002 | 0.003 | 0.04 |
| Striatum | Grafted | 0.085 | 0.082 | 0.003 | 0.002 | 0.24 |
|  | Ungrafted | 0.077 | 0.077 | 0.001 | 0.001 | 0.36 |
| Hypothalamus | Grafted | 0.081 | 0.076 | 0.002 | 0.002 | 0.02 |
|  | Ungrafted | 0.083 | 0.075 | 0.003 | 0.002 | 0.03 |
| Thalamus | Grafted | 0.084 | 0.087 | 0.001 | 0.001 | 0.00 |
|  | Ungrafted | 0.081 | 0.084 | 0.001 | 0.001 | 0.00 |
| Cerebellum | Grafted | 0.065 | 0.063 | 0.002 | 0.002 | 0.20 |
|  | Ungrafted | 0.066 | 0.063 | 0.002 | 0.003 | 0.20 |
| BrainStem | Grafted | 0.075 | 0.079 | 0.001 | 0.001 | 0.01 |
|  | Ungrafted | 0.074 | 0.077 | 0.001 | 0.002 | 0.02 |
| WhiteMatter | Grafted | 0.087 | 0.086 | 0.003 | 0.001 | 0.39 |
|  | Ungrafted | 0.084 | 0.083 | 0.002 | 0.001 | 0.44 |
| Ventricles | Grafted | 0.115 | 0.116 | 0.004 | 0.004 | 0.44 |
|  | Ungrafted | 0.111 | 0.116 | 0.005 | 0.003 | 0.21 |


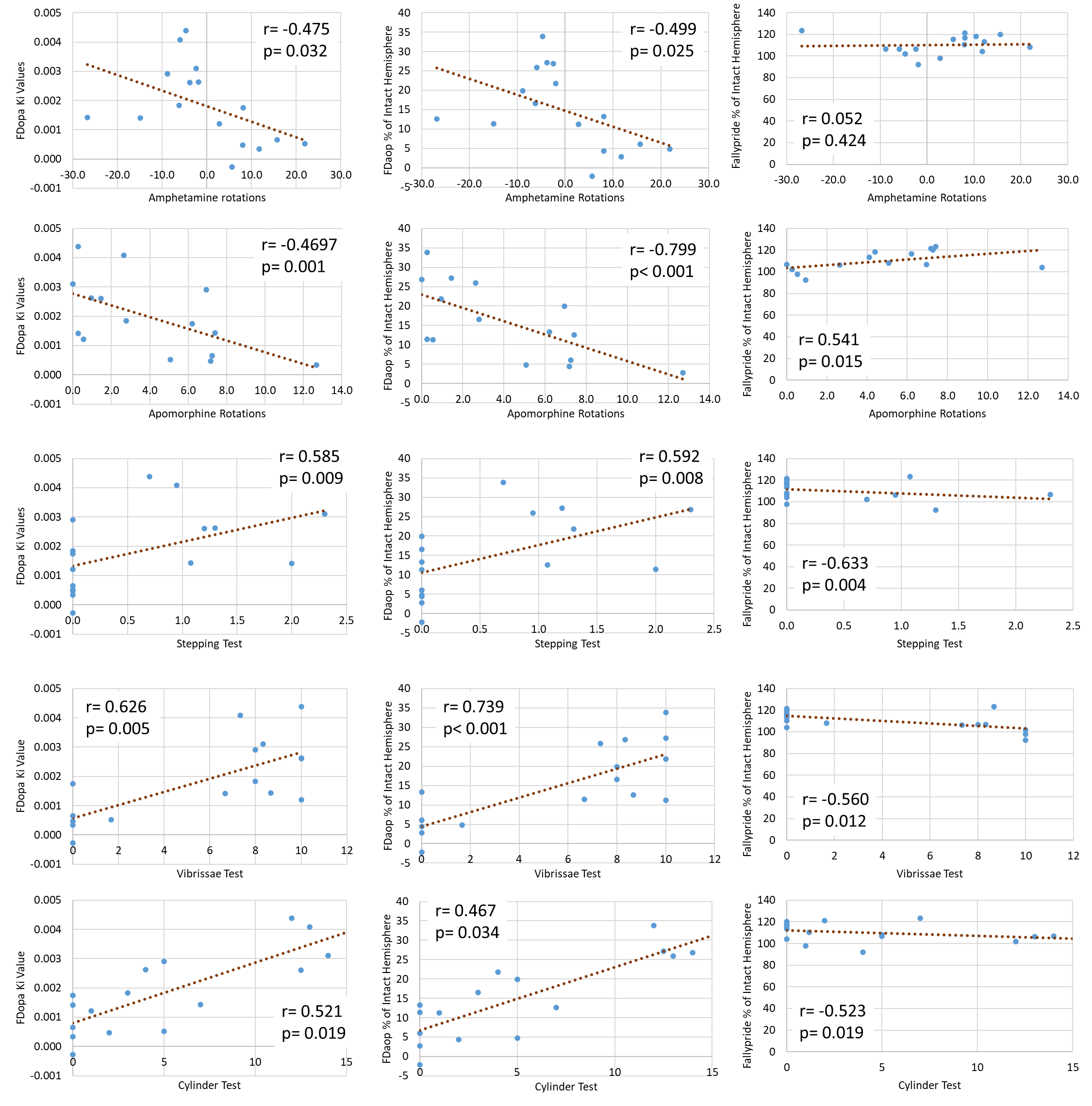


***Supplementary Figure 1: Correlations between hfVM PET data and behavioural data.*** [^18^F]Fluorodopa Ki raw values, [^18^F]Fluorodopa as a percentage of the intact hemisphere and [^18^F]Fallypride as a percentage of the intact hemisphere, were correlated with amphetamine-induced rotations, apomorphine-induced rotations, stepping test, vibrissae test, cylinder test.


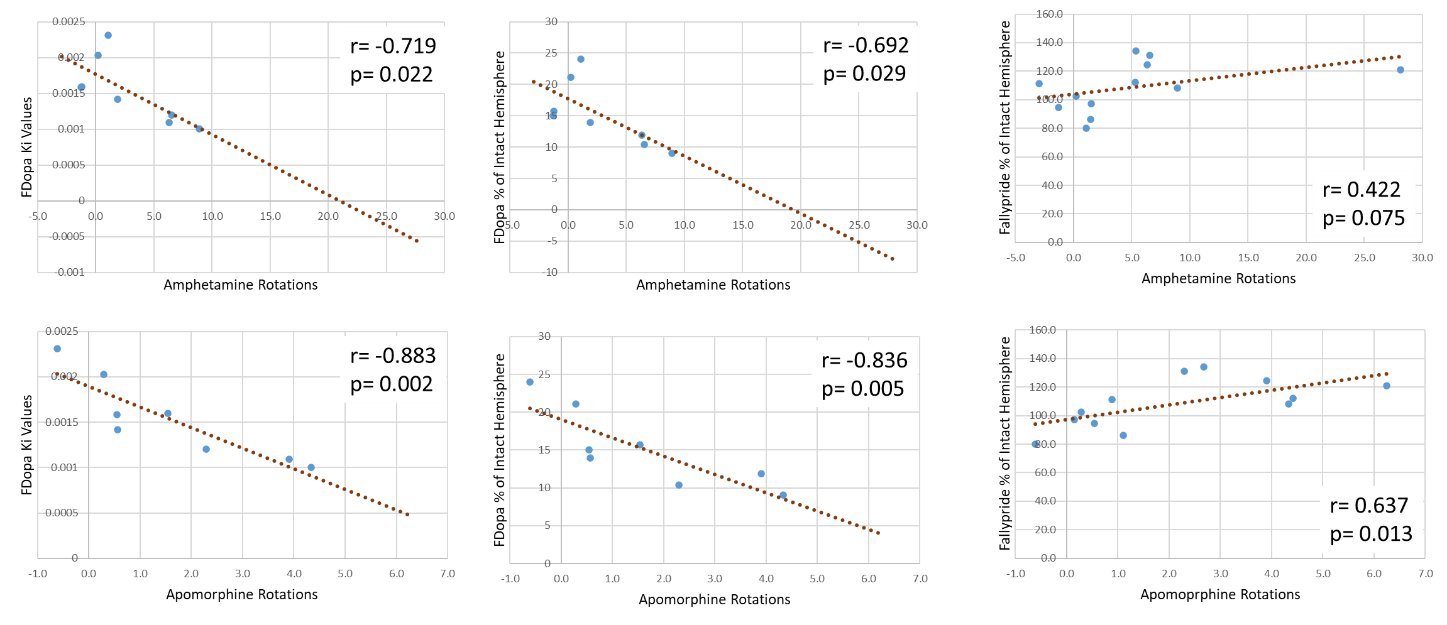


***Supplementary Figure 2: Correlations between hESC-DA PET data and behavioural data.*** [^18^F]Fluorodopa Ki raw values, [^18^F]Fluorodopa as a percentage of the intact hemisphere and [^18^F]Fallypride as a percentage of the intact hemisphere, were correlated with amphetamine-induced rotations and apomorphine-induced rotations.


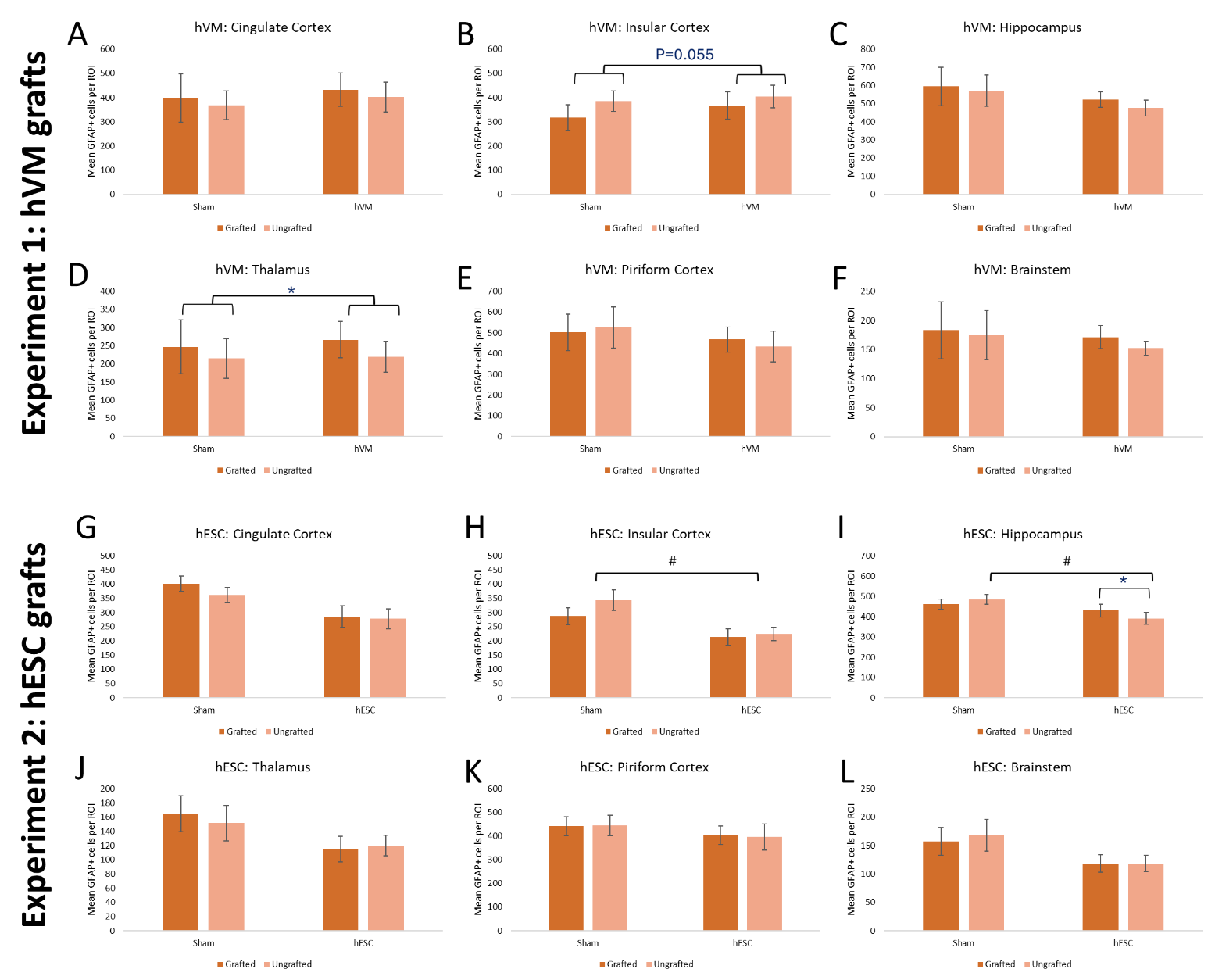


***Supplementary Figure 3: Stereological analysis of GFAP staining to identify changes in astrocytes.*** *In Experiment 1, the hfVM cohort, data were collected in (A) the cingulate cortex, (B) the insular cortex [Effect of hemisphere: F_1,14_=4.367, p=0.055], (C) the hippocampus, (D) the thalamus [Effect of hemisphere: F_1,14_=6.139, p<0.05], (E) the piriform cortex, (F) the brainstem. In Experiment 2, the hESC-DA grafted cohort, data were collected from (G) the cingulate cortex, (H) the insular cortex [Effect of group: F_1,17_=6.658, p=0.05], (I) the hippocampus [Group*Hemisphere interaction: F_1,17_=4.887, p<0.05; effect of hemisphere for hESC rats, p<0.05], (J) the thalamus, (K) the piriform cortex, (L) the brainstem. *=Effect of hemisphere. #=Effect of group. Data represent means and standard error of the means (S.E.M.s).*


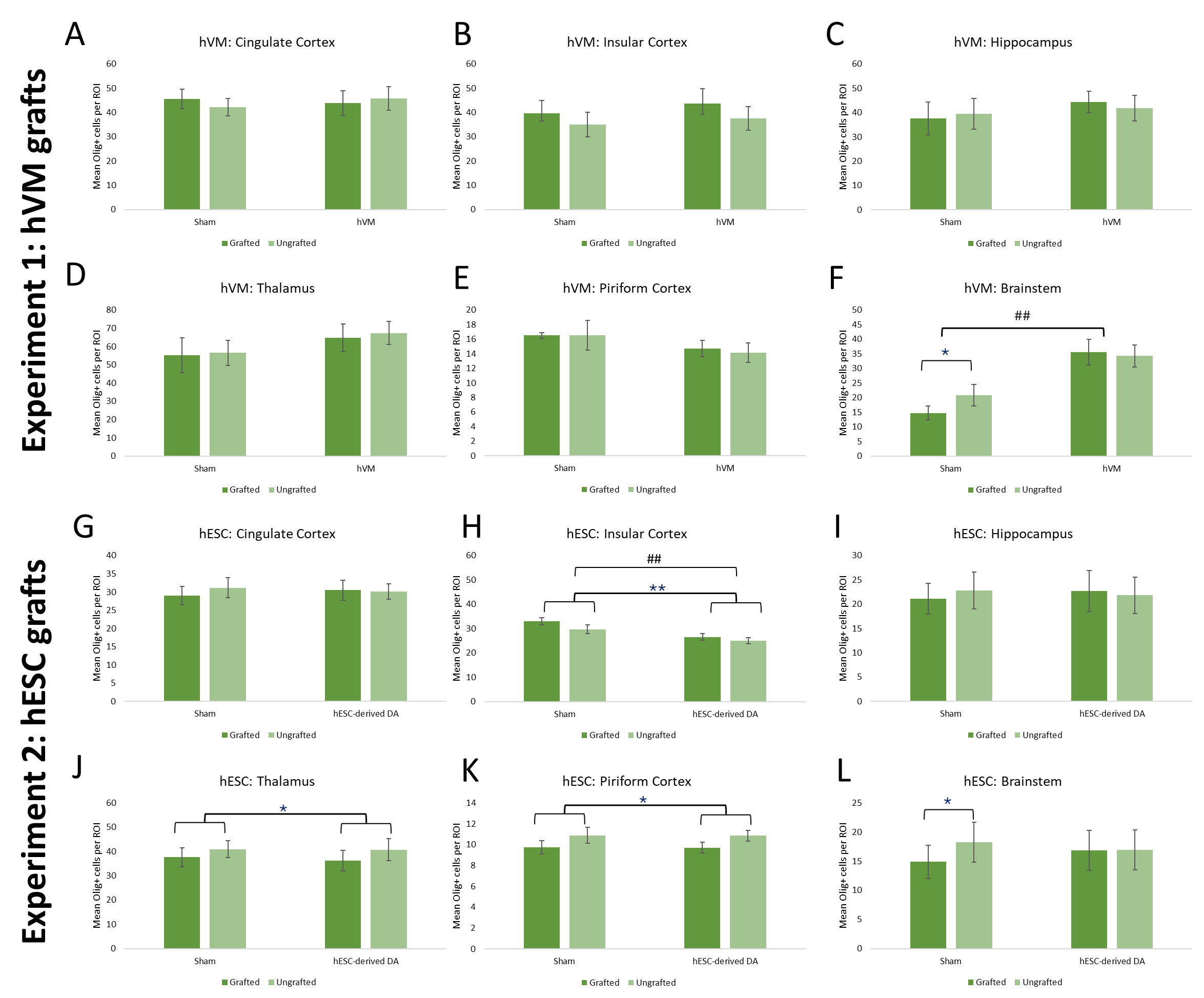
***Supplementary Figure 4: Stereological analysis of Olig2 staining to identify changes oligodendrocytes.*** *In Experiment 1, the hfVM cohort, data were collected in (A) the cingulate cortex, (B) the insular cortex, (C) the hippocampus, (D) the thalamus (E) the piriform cortex, (F) the brainstem [Effect of group: F_1,14_=8.826, p=0.01; Group*Hemisphere interaction: F_1,14_=9.003, p=0.01, effect of hemisphere in lesion rats, p<0.05]. In Experiment 2, the hESC-DA grafted cohort, data were collected from (G) the cingulate cortex, (H) the insular cortex [Effect of group: F_1,17_=8.619, p<0.01; effect of hemisphere: F_1,17_=9.458, p<0.01], (I) the hippocampus, (J) the thalamus effect of hemisphere: F_1,17_=5.749=X, p<0.05, (K) the piriform cortex effect of hemisphere: F_1,17_=4.525, p<0.05], (L) the brainstem ; Group*Hemisphere interaction: F_1,17_=6.188, p<0.05, effect of hemisphere in lesion rats, p<0.01]. *=Effect of hemisphere. #=Effect of group. Data represent means and standard error of the means (S.E.M.s).*

***
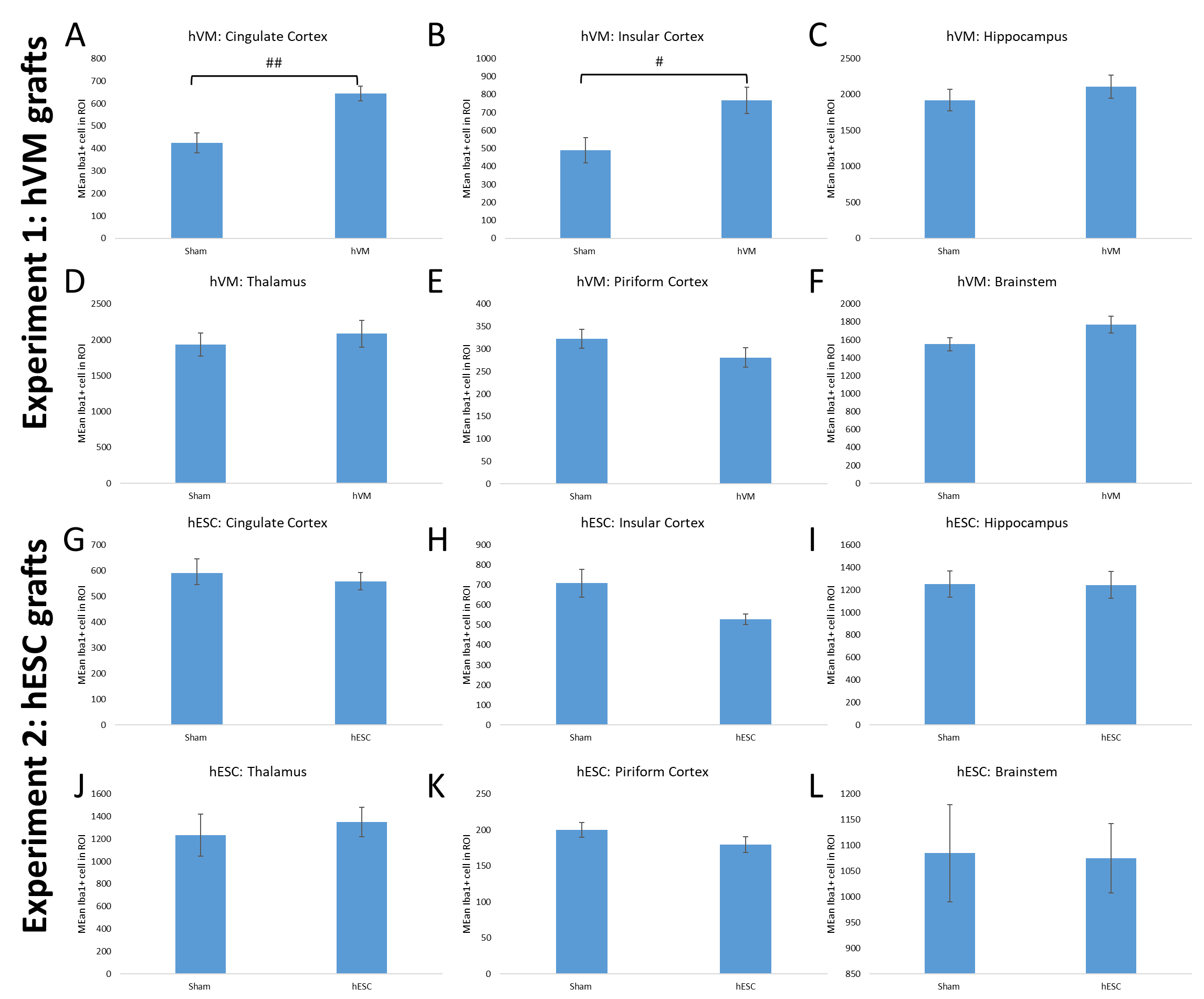
***

***Supplementary Figure 5: Analysis of Iba1 staining in the grafted hemisphere to identify changes microglia.*** *In Experiment 1, the hfVM cohort, data were collected in (A) the cingulate cortex [Group: F_1,14_=16.69, p=0.001], (B) the insular cortex [Group: F_1,14_=6.47, p<0.05], (C) the hippocampus, (D) the thalamus (E) the piriform cortex, (F) the brainstem. In Experiment 2, the hESC-DA grafted cohort, data were collected from (G) the cingulate cortex, (H) the insular cortex, (I) the hippocampus, (J) the thalamus, (K) the piriform cortex, (L) the brainstem. *=Effect of hemisphere. #=Effect of group. Data represent means and standard error of the means (S.E.M.s).*
